# Supplementary material for: Variability of amylose content and its correlation with the paste properties of cassava starch
Source: PLoS One. 2024 Oct 23;19(10):e0309619. doi: 10.1371/journal.pone.0309619 (PMC11498679; doi:10.1371/journal.pone.0309619)
Supplement: S1 Table — (DOCX) [file pone.0309619.s001.docx]

**Variability of amylose content and its correlation with the paste properties of cassava starch**

**Supplementary material**

**Table S1**. Origin of cassava genotypes with their classification regarding cyanogenic compound content (HCN), dry matter content (DMC), and starch content (**StC**).

| **Genotype** | **Population** | **HCN** | **DMC** | **StC** | **Origin** |
| --- | --- | --- | --- | --- | --- |
| 2019g-05-12 | Germplasm | Intermediate | High | High |  |
| 2019g-08-53 | Germplasm | Sweet | High | High |  |
| 2019g-08-58 | Germplasm | Sweet | High | Low |  |
| 2019g-08-63 | Germplasm | Sweet | High | Low |  |
| 2019g-08-67 | Germplasm | Sweet |  |  |  |
| 7027-1 | Improved |  |  |  | Brazil, BA |
| 7424-4 | Improved |  |  |  | Brazil, BA |
| 7803-2 | Improved |  |  |  | Brazil, BA |
| 7803-7 | Improved |  |  |  | Brazil, BA |
| 7878-18 | Improved |  |  |  | Brazil, BA |
| Aipim-Brazilia | Germplasm | Sweet | Low | High |  |
| Aipim-Guanambi | Germplasm | Sweet | High | Low |  |
| Baianinha | Germplasm | Sweet | Low |  |  |
| BGM-0018 | Germplasm | Sweet | High | High | Brazil, n.i. |
| BGM-0032 | Germplasm | Intermediate | High |  | Brazil, RN |
| BGM-0035 | Germplasm | Intermediate | High |  | Brazil, RN |
| BGM-0036 | Germplasm | Intermediate | Low |  | Brazil, BA |
| BGM-0048 | Germplasm | Intermediate | High |  | Brazil, BA |
| BGM-0061 | Germplasm | Bitter | High | High | Brazil, PA |
| BGM-0062 | Germplasm | Sweet | Low | High | Brazil, BA |
| BGM-0069 | Germplasm | Bitter | High | Low | Brazil, BA |
| BGM-0070 | Germplasm | Sweet | Low | High | Brazil, PA |
| BGM-0083 | Germplasm | Sweet | Low | High | Brazil, BA |
| BGM-0085 | Germplasm | Sweet | High | High | Brazil, SE |
| BGM-0093 | Germplasm | Bitter | High |  | Brazil, n.i. |
| BGM-0120 | Germplasm | Bitter | Low | Low | Brazil, BA |
| BGM-0122 | Germplasm | Bitter | High | High | Brazil, PA |
| BGM-0123 | Germplasm | Sweet | Low | Low | Brazil, BA |
| BGM-0133 | Germplasm | Intermediate | High |  | Brazil, BA |
| BGM-0137 | Germplasm | Bitter | Low |  | Brazil, BA |
| BGM-0138 | Germplasm | Bitter | Low |  | Brazil, BA |
| BGM-0162 | Germplasm | Bitter | High |  | Brazil, BA |
| BGM-0163 | Germplasm | Bitter |  |  | Brazil, BA |
| BGM-0169 | Germplasm | Bitter | High |  | Brazil, BA |
| BGM-0170 | Germplasm |  | High |  | Brazil, n.i. |
| BGM-0174 | Germplasm | Intermediate | High | High | Brazil, n.i. |
| BGM-0178 | Germplasm |  | High |  | Brazil, BA |
| BGM-0202 | Germplasm | Intermediate | Low | High | Brazil, BA |
| BGM-0236 | Germplasm | Bitter | High |  | Brazil, SP |
| BGM-0238 | Germplasm | Sweet | Low | High | Brazil, n.i. |
| BGM-0248 | Germplasm | Sweet | Low | Low | Brazil, BA |
| BGM-0249 | Germplasm | Sweet | High |  | Brazil, BA |
| BGM-0250 | Germplasm | Sweet |  |  | Brazil, BA |
| BGM-0252 | Germplasm | Sweet |  |  |  |
| BGM-0257 | Germplasm | Bitter | High | High | Brazil, BA |
| BGM-0264 | Germplasm | Bitter | High |  | Brazil, BA |
| BGM-0268 | Germplasm | Sweet | Low | High | Brazil, BA |
| BGM-0277 | Germplasm | Intermediate | Low |  | Brazil, SE |
| BGM-0284 | Germplasm | Intermediate | Low |  | Brazil, BA |
| BGM-0285 | Germplasm | Sweet | Low | Low | Brazil, n.i. |
| BGM-0286 | Germplasm | Sweet | Low | High | Brazil, n.i. |
| BGM-0288 | Germplasm | Sweet | Low | High | Brazil, BA |
| BGM-0297 | Germplasm | Sweet | High | Low | Colômbia, Valle |
| BGM-0306 | Germplasm | Intermediate | Low | High | Colômbia, Valle |
| BGM-0314 | Germplasm | Sweet | High | High | Colômbia, Valle |
| BGM-0316 | Germplasm |  | High |  | Colômbia, Valle |
| BGM-0338 | Germplasm | Sweet | High | High | Colômbia, Valle |
| BGM-0349 | Germplasm | Sweet | Low | High | Colômbia, Valle |
| BGM-0356 | Germplasm | Sweet | Low |  | Brazil, SP |
| BGM-0365 | Germplasm |  | Low |  | Brazil, CE |
| BGM-0368 | Germplasm | Sweet | High | High | Brazil, CE |
| BGM-0380 | Germplasm | Bitter | High | High | Brazil, CE |
| BGM-0390 | Germplasm | Bitter | Low | Low | Brazil, ES |
| BGM-0406 | Germplasm | Intermediate | High |  | Brazil, ES |
| BGM-0408 | Germplasm | Bitter | High | High | Brazil, n.i. |
| BGM-0411 | Germplasm | Bitter | High |  | Brazil, n.i. |
| BGM-0413 | Germplasm | Bitter | High |  | Brazil, MG |
| BGM-0414 | Germplasm | Intermediate | High |  | Brazil, SP |
| BGM-0422 | Germplasm | Sweet | Low |  | Brazil, SP |
| BGM-0425 | Germplasm | Intermediate | High |  | Colômbia, Valle |
| BGM-0470 | Germplasm | Bitter | High | High | Brazil, n.i. |
| BGM-0472 | Germplasm | Bitter | High | High | Brazil, RJ |
| BGM-0480 | Germplasm | Sweet | Low | High | Brazil, n.i. |
| BGM-0496 | Germplasm | Sweet | High | High | Brazil, ES |
| BGM-0545 | Germplasm | Sweet | High |  | Brazil, AL |
| BGM-0555 | Germplasm | Intermediate | Low |  | Brazil, n.i. |
| BGM-0562 | Germplasm | Sweet | Low |  | Brazil, BA |
| BGM-0583 | Germplasm | Intermediate | High | High | Brazil, AL |
| BGM-0606 | Germplasm | Sweet | High | High | Brazil, CE |
| BGM-0607 | Germplasm | Bitter | High |  | Brazil, n.i. |
| BGM-0636 | Germplasm | Intermediate | High | High | Brazil, CE |
| BGM-0659 | Germplasm | Intermediate | Low | High | Brazil, MA |
| BGM-0668 | Germplasm | Sweet | Low |  | Brazil, MG |
| BGM-0669 | Germplasm |  |  |  | Brazil, MA |
| BGM-0673 | Germplasm | Intermediate | High | Low | Brazil, MA |
| BGM-0695 | Germplasm | Intermediate |  |  |  |
| BGM-0703 | Germplasm |  |  |  | Brazil, AC |
| BGM-0714 | Germplasm | Sweet | High | High | Brazil, BA |
| BGM-0717 | Germplasm | Intermediate | High |  | Brazil, AC |
| BGM-0718 | Germplasm | Intermediate | High |  | Brazil, AC |
| BGM-0729 | Germplasm | Bitter | High | High | Brazil, BA |
| BGM-0737 | Germplasm | Sweet | Low | High | Brazil, ES |
| BGM-0760 | Germplasm | Sweet | Low | Low | Brazil, ES |
| BGM-0779 | Germplasm | Intermediate |  |  | Brazil, BA |
| BGM-0793 | Germplasm | Bitter | Low | High | Brazil, BA |
| BGM-0808 | Germplasm | Intermediate | High |  | Brazil, AL |
| BGM-0820 | Germplasm | Bitter | High |  | Brazil, SE |
| BGM-0831 | Germplasm | Sweet | Low | High | Brazil, SE |
| BGM-0850 | Germplasm | Sweet | High | High | Brazil, SE |
| BGM-0859 | Germplasm | Sweet | High | High | Brazil, PB |
| BGM-0873 | Germplasm | Intermediate | High |  | Colômbia, Valle |
| BGM-0878 | Germplasm | Bitter | High | High | Brazil, AM |
| BGM-0879 | Germplasm | Bitter | Low | High | Brazil, SC |
| BGM-0882 | Germplasm | Sweet | Low | Low | Colombia, Valle |
| BGM-0885 | Germplasm | Sweet | Low | Low | Colombia, Valle |
| BGM-0886 | Germplasm | Intermediate | Low | Low | Brazil, AM |
| BGM-0895 | Germplasm | Intermediate | High | High | Brazil, PA |
| BGM-0900 | Germplasm | Intermediate | High |  | Colombia, Valle |
| BGM-0901 | Germplasm | Sweet | High | High | Brazil, CE |
| BGM-0902 | Germplasm | Bitter | High | High | Brazil, PA |
| BGM-0914 | Germplasm | Bitter | High | High | Brazil, PA |
| BGM-0918 | Germplasm | Intermediate | High | High | Brazil, PA |
| BGM-0919 | Germplasm | Bitter | Low | High | Brazil, AM |
| BGM-0920 | Germplasm | Bitter | Low | Low | Brazil, AM |
| BGM-0925 | Germplasm | Intermediate | High |  | Brazil, DF |
| BGM-0941 | Germplasm | Bitter | High | Low | Brazil, AM |
| BGM-0942 | Germplasm | Intermediate | Low | High | Brazil, AM |
| BGM-0943 | Germplasm | Intermediate | Low | Low | Brazil, AM |
| BGM-0944 | Germplasm | Bitter | High |  | Brazil, AM |
| BGM-0946 | Germplasm | Bitter | Low | Low | Brazil, AM |
| BGM-0949 | Germplasm | Bitter | High | High | Brazil, AM |
| BGM-0951 | Germplasm | Intermediate | High | High | Brazil, AM |
| BGM-0952 | Germplasm | Bitter | Low | Low | Brazil, AM |
| BGM-0954 | Germplasm | Bitter | High | High | Brazil, AM |
| BGM-0955 | Germplasm |  |  |  | Brazil, AM |
| BGM-0957 | Germplasm | Intermediate | High |  | Brazil, PA |
| BGM-0959 | Germplasm | Intermediate | High |  | Brazil, AM |
| BGM-0962 | Germplasm | Bitter | High | High | Brazil, AM |
| BGM-0966 | Germplasm | Intermediate | High | High | Brazil, AM |
| BGM-0967 | Germplasm | Intermediate | High | Low | Brazil, AM |
| BGM-0971 | Germplasm | Intermediate | Low | High | Brazil, AM |
| BGM-0972 | Germplasm | Intermediate | High |  | Brazil, AM |
| BGM-0973 | Germplasm | Intermediate | High |  | Brazil, AM |
| BGM-0974 | Germplasm | Intermediate | Low | High | Brazil, AM |
| BGM-0975 | Germplasm | Bitter |  |  | Brazil, AM |
| BGM-0978 | Germplasm | Sweet | Low | High | Brazil, AM |
| BGM-0980 | Germplasm | Bitter | Low |  | Brazil, AM |
| BGM-0984 | Germplasm |  |  |  | Brazil, AM |
| BGM-0985 | Germplasm | Bitter | High |  | Brazil, AM |
| BGM-0991 | Germplasm | Sweet | Low | Low | Brazil, AM |
| BGM-0994 | Germplasm | Bitter |  |  | Brazil, AM |
| BGM-0995 | Germplasm | Intermediate | High | High | Colombia, Valle |
| BGM-0998 | Germplasm | Sweet | High | Low | Colombia, Valle |
| BGM-1023 | Germplasm | Sweet | High | High | Brazil, BA |
| BGM-1029 | Germplasm | Bitter | High |  | Brazil, BA |
| BGM-1034 | Germplasm | Intermediate | Low | High | Brazil, GO |
| BGM-1042 | Germplasm | Sweet | High | High | Brazil, CE |
| BGM-1044 | Germplasm | Sweet | High | High | Brazil, CE |
| BGM-1060 | Germplasm | Intermediate |  |  | Brazil, RN |
| BGM-1064 | Germplasm | Bitter | High |  | Brazil, PB |
| BGM-1103 | Germplasm | Sweet | High | High | Brazil, n.i. |
| BGM-1106 | Germplasm | Intermediate | High |  | Brazil, BA |
| BGM-1134 | Germplasm | Sweet | High | Low | Brazil, AM |
| BGM-1143 | Germplasm | Bitter | Low | Low | Brazil, MT |
| BGM-1148 | Germplasm | Bitter | High | High | Brazil, BA |
| BGM-1150 | Germplasm | Sweet | High | High | Brazil, BA |
| BGM-1152 | Germplasm | Bitter | High | High | Brazil, AM |
| BGM-1162 | Germplasm | Bitter | High | High | Brazil, n.i. |
| BGM-1164 | Germplasm | Bitter | Low | Low | Brazil, n.i. |
| BGM-1168 | Germplasm | Bitter | High | High | Brazil, n.i. |
| BGM-1189 | Germplasm | Intermediate | High |  | Brazil, PA |
| BGM-1213 | Germplasm | Bitter | High | High | Colombia, Valle |
| BGM-1224 | Germplasm | Intermediate | Low | High | Brazil, n.i. |
| BGM-1225 | Germplasm | Sweet | High | High | Brazil , n.i. |
| BGM-1251 | Germplasm | Sweet | High |  | Nigeria, n.i. |
| BGM-1252 | Germplasm | Bitter | High |  | Nigeria, n.i. |
| BGM-1253 | Germplasm | Intermediate | Low |  | Nigeria, n.i. |
| BGM-1265 | Germplasm | Intermediate | High |  | Brazil, PE |
| BGM-1288 | Germplasm | Sweet | High | High | Brazil, PE |
| BGM-1310 | Germplasm | Sweet | Low | Low | Brazil, CE |
| BGM-1313 | Germplasm | Sweet | High | High | Brazil, PE |
| BGM-1317 | Germplasm | Sweet | Low | High | Brazil, PE |
| BGM-1332 | Germplasm | Sweet | High | High | Brazil, PB |
| BGM-1344 | Germplasm | Intermediate |  |  | Brazil, PB |
| BGM-1355 | Germplasm | Intermediate | Low | Low | Brazil, PB |
| BGM-1362 | Germplasm | Intermediate | Low | High | Brazil, PB |
| BGM-1367 | Germplasm | Sweet |  |  | Brazil, PB |
| BGM-1377 | Germplasm | Sweet | Low | Low | Brazil, PB |
| BGM-1390 | Germplasm | Bitter | High | High | Brazil, RN |
| BGM-1397 | Germplasm | Intermediate | Low | High | Brazil, RN |
| BGM-1432 | Germplasm | Sweet | High | High | Brazil, PE |
| BGM-1447 | Germplasm | Intermediate | High | High | Brazil, BA |
| BGM-1454 | Germplasm | Intermediate | High | Low | Brazil, PR |
| BGM-1483 | Germplasm | Sweet | High | High | Brazil, BA |
| BGM-1497 | Germplasm | Sweet | High | High | Brazil, BA |
| BGM-1498 | Germplasm | Intermediate | Low | Low | Brazil, BA |
| BGM-1502 | Germplasm | Sweet | Low | High | Brazil, BA |
| BGM-1502-M | Germplasm | Sweet | Low | High |  |
| BGM-1503 | Germplasm | Bitter | High | High | Brazil, BA |
| BGM-1518 | Germplasm | Bitter | High | High | Brazil, BA |
| BGM-1586 | Germplasm | Bitter | High | High | Brazil, PI |
| BGM-1659 | Germplasm | Bitter | Low | Low | Brazil, BA |
| BGM-1660 | Germplasm | Sweet | High | Low | Brazil, BA |
| BGM-1677 | Germplasm | Sweet | High | High | Brazil, PA |
| BGM-1679 | Germplasm | Sweet | Low | Low | Brazil, PA |
| BGM-1683 | Germplasm | Bitter | High | Low | Brazil, BA |
| BGM-1689 | Germplasm | Sweet | High |  | Brazil, BA |
| BGM-1693 | Germplasm | Intermediate | Low |  | Brazil, BA |
| BGM-1703 | Germplasm | Bitter | High | High | Brazil, AM |
| BGM-1704 | Germplasm | Bitter |  |  | Brazil, AM |
| BGM-1706 | Germplasm | Sweet | Low | Low | Brazil, AM |
| BGM-1709 | Germplasm | Bitter | Low | High | Brazil, AM |
| BGM-1713 | Germplasm | Bitter |  |  | Brazil, AM |
| BGM-1718 | Germplasm | Bitter | Low | Low | Brazil, AM |
| BGM-1719 | Germplasm |  | Low | High | Brazil, MA |
| BGM-1725 | Germplasm | Bitter | Low | Low | Brazil, BA |
| BGM-1735 | Germplasm |  | High | High | Brazil, MA |
| BGM-1739 | Germplasm | Bitter | High | Low | Brazil, MA |
| BGM-1741 | Germplasm | Bitter | High | High | Brazil, MA |
| BGM-1745 | Germplasm | Bitter | High | High | Brazil, MA |
| BGM-1747 | Germplasm |  | Low | High | Brazil, MA |
| BGM-1752 | Germplasm | Bitter | High | High | Brazil, MA |
| BGM-1754 | Germplasm | Bitter | High |  | Brazil, MA |
| BGM-1757 | Germplasm | Sweet | Low |  | Brazil, MA |
| BGM-1759 | Germplasm | Bitter | Low | High | Brazil, MA |
| BGM-1762 | Germplasm | Sweet | High | High | Brazil, MA |
| BGM-1772 | Germplasm | Sweet |  |  | Brazil, MA |
| BGM-1779 | Germplasm | Bitter | High | High | Brazil, MA |
| BGM-1780 | Germplasm | Bitter | High | High | Brazil, MA |
| BGM-1783 | Germplasm | Bitter | High | High | Brazil, MA |
| BGM-1784 | Germplasm | Bitter | High | High | Brazil, MA |
| BGM-1794 | Germplasm | Bitter | High | High | Brazil, MA |
| BGM-1821 | Germplasm | Sweet | High | High | Brazil, BA |
| BGM-1834 | Germplasm | Sweet | Low | Low | Brazil, PA |
| BGM-1873 | Germplasm | Intermediate | Low |  | Brazil, MT |
| BGM-1880 | Germplasm | Sweet | Low | High | Brazil, BA |
| BGM-1884 | Germplasm |  |  |  | Brazil, AM |
| BGM-1942 | Germplasm | Intermediate | Low |  | Brazil, PA |
| BGM-1957 | Germplasm | Bitter | Low | Low | Brazil, AM |
| BGM-2034 | Germplasm |  | Low | Low | Brazil, MA |
| BGM-2038 | Germplasm |  | High | High | Brazil, BA |
| BGM-2047 | Germplasm | Intermediate | Low | High | Colombia, Valle |
| BGM-2057 | Germplasm | Sweet | Low | Low | Brazil, RS |
| BGM-2066 | Germplasm |  | Low | Low | Brazil, RS |
| BGM-2074 | Germplasm | Sweet | High | Low | Brazil, RS |
| BGM-2094 | Germplasm | Intermediate | High |  | Brazil, PR |
| BGM-2095 | Germplasm | Bitter | High | High | Brazil, BA |
| BGM-2097 | Germplasm | Sweet | Low | High | Brazil, PR |
| BGM-2100 | Germplasm |  |  |  | Brazil, PR |
| BGM-2102 | Improved | Intermediate | High |  | Brazil, PR |
| BGM-2105 | Improved | Intermediate | Low | Low | Brazil, PR |
| BGM-2119 | Germplasm | Intermediate | Low |  | Brazil, PR |
| BGM-2124 | Improved | Intermediate | High | High | Brazil, BA |
| BGM-2127 | Germplasm |  | Low | Low | Brazil, DF |
| BGM-2151 | Germplasm | Bitter | High | High | Brazil, PE |
| BGM-2160 | Germplasm | Sweet | High | High | Brazil, ES |
| BGM-2164 | Germplasm | Intermediate | High |  | Brazil, ES |
| BGM-2167 | Germplasm | Intermediate | Low | Low | Brazil, ES |
| BGM-2187 | Germplasm |  | Low | Low | Brazil, PE |
| BGM-2209 | Germplasm | Sweet | High | High | Brazil, PE |
| BGM-2265 | Germplasm | Intermediate | Low | High | Brazil, PE |
| BGM-2270 | Germplasm |  | Low |  | Brazil, PE |
| BGM-2276 | Germplasm | Sweet | Low | High | Brazil, PE |
| BGM-2339 | Germplasm | Bitter | High |  |  |
| BGM-2345 | Germplasm | Sweet | High |  | Colombia, Valle |
| BGM-2358 | Germplasm |  | High | High |  |
| BR-11-34-41 | Improved | Sweet | Low | High | Brazil, BA |
| BR-11-34-45 | Improved | Intermediate | High | High | Brazil, BA |
| BR-11-34-64 | Improved | Sweet | Low | High | Brazil, BA |
| BR-18-F2wx-113-07 | Improved | Intermediate | High | Low | Brazil, BA |
| BR-19F1wx-020-12 | Improved | Bitter | High |  | Brazil, BA |
| BR-19F1wx-054-29 | Improved | Intermediate | Low |  | Brazil, BA |
| BR-19F2wx-045-8 | Improved | Bitter | High |  | Brazil, BA |
| BR-19F2wx-204-15 | Improved | Sweet | Low |  | Brazil, BA |
| BR-19F2wx-356-2 | Improved | Intermediate | Low |  | Brazil, BA |
| BR-20F2Polwx-012-01 | Improved | Sweet | High |  | Brazil, BA |
| BR-20F2Polwx-021-06 | Improved |  |  |  | Brazil, BA |
| BRS-396 | Improved | Sweet | Low | Low |  |
| BRS-399 | Improved | Sweet | Low | Low |  |
| BRS-CS01 | Improved | Bitter | Low | Low |  |
| BRS-Dourada | Improved | Sweet | Low | Low |  |
| BRS-Formosa | Improved | Bitter | High | Low |  |
| BRS-Gema-de-Ovo | Improved | Sweet | High | High | Brazil, PA |
| BRS-Jari | Improved | Sweet | Low | Low | Brazil, BA |
| BRS-Kiriris | Improved | Sweet | High | High |  |
| BRS-Mulatinha | Improved | Bitter | High | High | Brazil, BA |
| BRS-Poti-Branca | Improved | Bitter | Low | High | Brazil, BA |
| BRS-Tapioqueira | Improved | Bitter | High |  | Brazil, BA |
| Clone-7737-1 | Improved |  |  |  |  |
| CPAFRO-09 | Germplasm |  |  |  |  |
| Maragojipe | Improved | Sweet | High | Low |  |
| Uberlandia-2 | Improved | Intermediate |  | Low |  |
