# Supplementary material for: Variability of amylose content and its correlation with the paste properties of cassava starch
Source: PLoS One. 2024 Oct 23;19(10):e0309619. doi: 10.1371/journal.pone.0309619 (PMC11498679; doi:10.1371/journal.pone.0309619)
Supplement: Attachment — Submitted filename: PONE-D-24-09179_reviewed.pdf [file pone.0309619.s003.pdf]

PLOS ONE

Variability of amylose content and its correlation with the paste properties of cassava starch

--Manuscript Draft--

**Manuscript Number:**	PONE-D-24-09179
**Article Type:**	Research Article
**Full Title:**	Variability of amylose content and its correlation with the paste properties of cassava starch
**Short Title:**	amylose content × paste properties in cassava
**Corresponding Author:**	Eder Jorge de Oliveira, PhD<br>EMBRAPA Centro Nacional de Pesquisa de Mandioca e Fruticultura Tropical<br>Cruz das Almas, Bahia BRAZIL
**Keywords:**	germplasm; Manihot esculenta Crantz; root quality; breeding.
**Abstract:**	The amylose content can significantly impact the diverse industrial applications of cassava starch. This study aimed to assess the variability of cassava germplasm concerning amylose content and other attributes pertinent to root quality, alongside its correlation with paste properties. Starch extracted from 281 genotypes underwent evaluation for amylose content, with additional analysis of parameters such as pasting temperature, time to peak viscosity (TPV), viscosity breakdown (BrD), retrogradation tendency, and maximum, minimum, and final viscosities. The genotypes exhibited considerable variation in dry matter content (ranging from 27.06% to 41.02%), starch content (from 14.61% to 25.67%), cyanogenic compounds (1.77 to 7.81), and amylose content (0.05% to 33.23%). High phenotypic variability in paste properties was observed, alongside a low residual effect for most traits, resulting in high broad-sense heritabilities (>0.95). Strong correlations of significant magnitude (>0.80) were found between parameters such as peak viscosity × viscosity breakdown, minimum viscosity × final viscosity, and final viscosity × retrogradation tendency. Moderate correlations were also identified, such as between dry matter content × starch content (0.56). While positive, correlations between amylose content and paste properties were of low magnitude (ranging from 0.13 to 0.35), except for TPV and BrD. Principal component discriminant analysis clustered the germplasm into six distinct groups based on root quality and paste properties, with most improved genotypes falling into two clusters characterized by high starch and dry matter contents. This study underscores the necessity of simultaneous evaluation of amylose content and paste properties in the selection process. Additionally, clustering cassava genotypes proves beneficial in identifying those that fulfill specific requirements in industrial and breeding applications.
**Order of Authors:**	Natalia Rocha Ribeiro
	Massaine Bandeira e Sousa
	Luciana Alves de Oliveira
	Eder Jorge de Oliveira, PhD
**Additional Information:**	
**Question**	**Response**
**Financial Disclosure**<br><br>Enter a financial disclosure statement that describes the sources of funding for the work included in this submission. Review the submission guidelines for detailed requirements. View published research articles from *PLOS ONE* for specific examples.	Yes

Powered by Editorial Manager® and ProduXion Manager® from Aries Systems Corporation

This statement is required for submission and **will appear in the published article** if the submission is accepted. Please make sure it is accurate.<br><br>**Funded studies**<br>Enter a statement with the following details:<br>• Initials of the authors who received each award<br>• Grant numbers awarded to each author<br>• The full name of each funder<br>• URL of each funder website<br>• Did the sponsors or funders play any role in the study design, data collection and analysis, decision to publish, or preparation of the manuscript?<br><br>Did you receive funding for this work?	
Please add funding details.<br>as follow-up to "**Financial Disclosure**<br><br>Enter a financial disclosure statement that describes the sources of funding for the work included in this submission. Review the submission guidelines for detailed requirements. View published research articles from *PLOS ONE* for specific examples.<br><br>This statement is required for submission and **will appear in the published article** if the submission is accepted. Please make sure it is accurate.<br><br>**Funded studies**<br>Enter a statement with the following details:<br>• Initials of the authors who received each award<br>• Grant numbers awarded to each author<br>• The full name of each funder<br>• URL of each funder website<br>• Did the sponsors or funders play any role in the study design, data collection and analysis, decision to publish, or preparation of the manuscript?	Natalia Rocha Ribeiro: CAPES (Coordenação de Aperfeiçoamento de Pessoal de Nível Superior). Grant number: 88887.641163/2021-00; Massaine Bandeira e Sousa: Empresa Brasileira de Pesquisa Agropecuária. Grant number: 20.18.01.012.00.00; Eder Jorge de Oliveira: CNPq (Conselho Nacional de Desenvolvimento Científico e Tecnológico). Grant number: 409229/2018-0, 442050/2019-4 and 303912/2018-9; Eder Jorge de Oliveira: FAPESB (Fundação de Amparo à Pesquisa do Estado da Bahia). Grant number: Pronem 15/2014. This work was partially funded by UK's Foreign, Commonwealth & Development Office (FCDO) and the Bill & Melinda Gates Foundation. Grant INV-007637. Under the grant conditions of the Foundation, a Creative Commons Attribution 4.0 Generic License has already been assigned to the Author Accepted Manuscript version that might arise from this submission. The funder provided support in the form of fellowship and funds for the research, but did not have any additional role in the study design, data collection and analysis, decision to publish, nor preparation of the manuscript.

Powered by Editorial Manager® and ProduXion Manager® from Aries Systems Corporation

Did you receive funding for this work?"	
Please select the country of your main research funder (please select carefully as in some cases this is used in fee calculation).<br>as follow-up to "**Financial Disclosure**<br><br>Enter a financial disclosure statement that describes the sources of funding for the work included in this submission. Review the submission guidelines for detailed requirements. View published research articles from *PLOS ONE* for specific examples.<br><br>This statement is required for submission and **will appear in the published article** if the submission is accepted. Please make sure it is accurate.<br><br>**Funded studies**<br>Enter a statement with the following details:<br>• Initials of the authors who received each award<br>• Grant numbers awarded to each author<br>• The full name of each funder<br>• URL of each funder website<br>• Did the sponsors or funders play any role in the study design, data collection and analysis, decision to publish, or preparation of the manuscript?<br><br>Did you receive funding for this work?"	BRAZIL - BR
**Competing Interests**<br><br>Use the instructions below to enter a competing interest statement for this submission. On behalf of all authors, disclose any competing interests that could be perceived to bias this work—acknowledging all financial support and any other relevant financial or non-financial competing interests.<br><br>This statement is required for submission	The authors have declared that no competing interests exist.

Powered by Editorial Manager® and ProduXion Manager® from Aries Systems Corporation

and **will appear in the published article** if the submission is accepted. Please make sure it is accurate and that any funding sources listed in your Funding Information later in the submission form are also declared in your Financial Disclosure statement.<br><br>View published research articles from *PLOS ONE* for specific examples.<br><br>**NO authors have competing interests**<br><br>Enter: *The authors have declared that no competing interests exist.*<br><br>**Authors with competing interests**<br><br>Enter competing interest details beginning with this statement:<br><br>*I have read the journal's policy and the authors of this manuscript have the following competing interests: [insert competing interests here]*<br><br>* typeset	
**Ethics Statement**<br><br>Enter an ethics statement for this submission. This statement is required if the study involved:<br><br>• Human participants<br>• Human specimens or tissue<br>• Vertebrate animals or cephalopods<br>• Vertebrate embryos or tissues<br>• Field research<br><br>Write "N/A" if the submission does not require an ethics statement.<br><br>General guidance is provided below. Consult the submission guidelines for detailed instructions. **Make sure that all information entered here is included in the Methods section of the manuscript.**	N/A

**Format for specific study types**<br><br>**Human Subject Research (involving human participants and/or tissue)**<br>• Give the name of the institutional review board or ethics committee that approved the study<br>• Include the approval number and/or a statement indicating approval of this research<br>• Indicate the form of consent obtained (written/oral) or the reason that consent was not obtained (e.g. the data were analyzed anonymously)<br><br>**Animal Research (involving vertebrate animals, embryos or tissues)**<br>• Provide the name of the Institutional Animal Care and Use Committee (IACUC) or other relevant ethics board that reviewed the study protocol, and indicate whether they approved this research or granted a formal waiver of ethical approval<br>• Include an approval number if one was obtained<br>• If the study involved *non-human primates*, add *additional details* about animal welfare and steps taken to ameliorate suffering<br>• If anesthesia, euthanasia, or any kind of animal sacrifice is part of the study, include briefly which substances and/or methods were applied<br><br>**Field Research**<br><br>Include the following details if this study involves the collection of plant, animal, or other materials from a natural setting:<br>• Field permit number<br>• Name of the institution or relevant body that granted permission	
**Data Availability**<br><br>Authors are required to make all data underlying the findings described fully available, without restriction, and from the time of publication. PLOS allows rare exceptions to address legal and ethical concerns. See the PLOS Data Policy and FAQ for detailed information.	Yes - all data are fully available without restriction

Powered by Editorial Manager® and ProduXion Manager® from Aries Systems Corporation

A Data Availability Statement describing where the data can be found is required at submission. Your answers to this question constitute the Data Availability Statement and **will be published in the article**, if accepted.<br><br>**Important:** Stating 'data available on request from the author' is not sufficient. If your data are only available upon request, select 'No' for the first question and explain your exceptional situation in the text box.<br><br>Do the authors confirm that all data underlying the findings described in their manuscript are fully available without restriction?	
**Describe where the data may be found in full sentences. If you are copying our sample text, replace any instances of XXX with the appropriate details.**<br><br>• If the data are **held or will be held in a public repository**, include URLs, accession numbers or DOIs. If this information will only be available after acceptance, indicate this by ticking the box below. For example: *All XXX files are available from the XXX database (accession number(s) XXX, XXX.).*<br>• If the data are all contained **within the manuscript and/or Supporting Information files**, enter the following: *All relevant data are within the manuscript and its Supporting Information files.*<br>• If neither of these applies but you are able to provide **details of access elsewhere**, with or without limitations, please do so. For example:<br><br>*Data cannot be shared publicly because of [XXX]. Data are available from the XXX Institutional Data Access / Ethics Committee (contact via XXX) for researchers who meet the criteria for access to confidential data.*<br><br>*The data underlying the results presented in the study are available from (include the name of the third party*	All relevant data are within the manuscript and its Supporting Information files.

and contact information or URL).<br>• This text is appropriate if the data are owned by a third party and authors do not have permission to share the data.<br><br>* typeset	
Additional data availability information:	

Manuscript

Click here to access/download;Manuscript;Ribeiro et al_PlosOne_2024 manuscript.docx

Variability of amylose content and its correlation with the paste properties of cassava starch

Natalia Rocha Ribeiro[1], Massaine Bandeira e Sousa[2], Luciana Alves de Oliveira[2], Eder Jorge de Oliveira[2*]

[1]Universidade Federal do Recôncavo da Bahia, Cruz das Almas, Bahia, 44380-000, Brazil

[2]Embrapa Mandioca e Fruticultura, Cruz das Almas, Bahia, 44380-000, Brazil

Running title: amylose content × paste properties in cassava

*Corresponding author:

E-mail:eder.oliveira@embrapa.br

Natalia Rocha Ribeiro - ORCID: 0000-0001-9832-3235

Massaine Bandeira Sousa - ORCID: 0000-0001-7887-9543

Luciana Alves de Oliveira - ORCID: 0000-0002-6601-5884

Eder Jorge De Oliveira - ORCID: 0000-0001-8992-7459

Abstract:

The amylose content can significantly impact the diverse industrial applications of cassava starch. This study aimed to assess the variability of cassava germplasm concerning amylose content and other attributes pertinent to root quality, alongside its correlation with paste properties. Starch extracted from 281 genotypes underwent evaluation for amylose content, with additional analysis of parameters such as pasting temperature, time to peak viscosity (TPV), viscosity breakdown (BrD), retrogradation tendency, and maximum, minimum, and final viscosities. The genotypes exhibited considerable variation in dry matter content (ranging from 27.06% to 41.02%), starch content (from 14.61% to 25.67%), cyanogenic compounds (1.77 to 7.81), and amylose content (0.05% to 33.23%). High phenotypic variability in paste properties was observed, alongside a low residual effect for most traits, resulting in high broad-sense heritabilities (>0.95). Strong correlations of significant magnitude (>0.80) were found between parameters such as peak viscosity × viscosity breakdown, minimum viscosity × final viscosity, and final viscosity × retrogradation tendency. Moderate correlations were also identified, such as between dry matter content × starch content (0.56). While positive, correlations between amylose content and paste properties were of low magnitude (ranging from 0.13 to 0.35), except for TPV and BrD. Principal component discriminant analysis clustered the germplasm into six distinct groups based on root quality and paste properties, with most improved genotypes falling into two clusters characterized by high starch and dry matter contents. This study underscores the necessity of simultaneous evaluation of amylose content and paste properties in the selection process. Additionally, clustering cassava genotypes proves beneficial in identifying those that fulfill specific requirements in industrial and breeding applications.

Keywords: germplasm; *Manihot esculenta* Crantz; root quality; breeding.

1. Introduction

Cassava (*Manihot esculenta* Crantz) is a staple crop, and therefore, a food security specie for millions of people in tropical and subtropical regions worldwide, ranking as the fourth most important staple food after rice, wheat, and maize (1,2). The species holds significant economic importance as one of the primary sources of starch, alongside maize, potato, and rice (3,4), displaying reduced syneresis and a neutral flavor, thus providing a competitive edge in the food industry over cereal starches (5).

Industrial applications of starch rely on paste properties, including gelatinization (6–8), which transforms starch into a viscous paste with increasing temperature. Understanding the optimal water quantity and temperature is crucial, varying according to the type of starch (9).

The viscosity of starch suspensions is assessed using viscoamylographs such as Brabender and RVA. Peak viscosity of cassava starch (ranging between 146-1505 cP), breakdown (ranging between 28-859 cP), and retrogradation tendency (ranging between -702 to 273 cP) are critical points, influencing the quality and performance of starch in various industrial and culinary applications (10).

Santos et al. (11) reported that cassava starch exhibits a lower gelatinization temperature and reduced retrogradation tendency compared to cereal starches. This characteristic implies greater stability and a diminished likelihood of clumping or stickiness when used as a thickener or stabilizer in food products. Additionally, cassava starch is noted for its resistance to breakdown during industrial processing, resulting in final products with improved texture and appearance (10). Carmo et al. (5) and Sakurai et al. (12) further highlight various advantages of cassava starch, including its suitability as a gluten-free alternative for individuals with celiac disease and its high clarity when forming pastes. Moreover, the high purity of cassava starch makes it a preferred choice for use in the food industry.

Within the same species, there exist variations in paste properties and amylose content, offering opportunities for the selection of new varieties to be explored for various industrial purposes (13). Generally, some researchers suggest that starches with higher amylose contents are better suited in the manufacture of biofilms (21), in the production of adhesives and as binder (34) while those with lower amylose content are more commonly employed in the frozen food (11). Therefore, alongside numerous chemical modifications targeting specific properties, genetic variability can be leveraged in the development of cultivars with naturally modified starches.

In recent years, numerous studies have documented variability in paste properties among cassava starches (10,14–19). Sánchez et al. (10) provided a comprehensive assessment by evaluating approximately

4,000 cassava genotypes from the International Center for Tropical Agriculture (CIAT), revealing significant variability in peak viscosity (146 – 1,505 cP), breakdown (28.1 – 859 cP), and retrogradation tendency (-702–273 cP) within the CIAT germplasm. In another recent study in Brazil, Santos et al. (17) assessed over 1000 cassava germplasm accessions and reported distinct variations in peak viscosity (3,279 - 6,459 cP), breakdown (134 - 848 cP), and retrogradation tendency (290- 3,681 cP). Generally, starches with lower retrogradation tendencies are preferred as gelling agents in refrigerated and frozen foods, whereas those with higher swelling power (temperature ranging from 62.6 to 70.1 °C) are recommended for use as thickeners and emulsifiers in food products (16). Thus, the considerable variability present in cassava germplasm facilitates the selection and development of varieties with added value for various agri-food purposes.

The ability of starch granules to form a paste post-gelatinization is primarily attributed to the presence of amylose (20,21), which plays a pivotal role in determining the physicochemical properties of starch, including its paste characteristics (6,8). Normal or native cassava starches typically contain an average of 21% amylose, with a range spanning from 13% to 29% (10,20). While cassava mutants with starch comprised entirely of amylopectin (waxy starch) or with smaller granules containing approximately 30% amylose have been identified (22), there are no documented instances of cassava with notably high amylose content (>40%).

In starches with elevated amylose content, gelatinization tends to occur more rapidly, resulting in shorter cooking times for roots (20). Significant correlations between paste properties and amylose content have been established in various crops, including red rice (23), maize (24), potato (25), and sorghum (26). Karakelle et al. (24) noted that starches with high amylose content exhibit substantially lower viscosity values and higher pasting temperatures compared to those with lower amylose content. Conversely, in sorghum, amylose content displayed negative correlations with peak viscosity or maximum viscosity and positive correlations with retrogradation tendency and cold paste viscosity (26). These findings suggest that optimizing the amylose/amylopectin ratio can be achieved without the need for modifying starch properties to attain products with the desired characteristics.

Despite the crucial role of amylose in starch functionality, the correlation between paste properties and amylose content in cassava remains underexplored but is vital for guiding genetic improvement efforts. Therefore, comprehensive studies involving diverse populations exhibiting significant amylose content variations are imperative for accurately delineating its relationship with paste properties, with the goal of

developing more promising industrial starch varieties (6). Thus, this study aims to analyze the genetic diversity within the cassava germplasm bank concerning amylose content, investigating its association with starch paste properties in a population showcasing substantial variations in root yield and attributes.

2. Material and methods

2.1 Evaluation of cassava root quality-associated traits

A total of 30 trials evaluating clones from the Embrapa breeding program were conducted in the cities of Cruz das Almas, Laje, and Valença in Bahia, Brazil, from 2011 to 2022. Detailed information regarding the soil type at each evaluation site is presented in Table 1. According to the Köppen classification, the climate in the region is type Af, indicating a hot and humid tropical climate, with an average annual precipitation ranging from 1,200 to 1,500 mm, highest rainfall occurring from March to July, average annual temperature of 24.5 ºC, and average relative humidity of approximately 80%.

Table 1. Information about the locations where trials for evaluating root quality traits were conducted, from 2011 to 2022.

Trial	City / state	Altitude	Soil type	N# trials
CNPMF	Cruz das Almas, BA	215	Yellow Latosol	14
CNPMF Area 2	Cruz das Almas, BA	215	Yellow Latosol	1
Coopamido	Laje, BA	180	Red Yellow Latosol	4
Novo Horizonte	Laje, BA	180	Red Yellow Latosol	2
Novo Rumo	Laje, BA	180	Red Yellow Latosol	1
UFRB-PP1	Cruz das Almas, BA	215	Yellow Latosol	5
UFRB-Candial	Cruz das Almas, BA	215	Yellow Latosol	3

In total, 281 genotypes were evaluated in the field (Table S1) using an augmented block design, with plots consisting of two rows with eight to ten plants each (totaling 16 to 20 plants per plot), spaced 0.90 m between rows and 0.80 m between plants. Between 4 and 34 improved cultivars from Embrapa and local cultivars were planted as common checks in different augmented blocks (ranging from 5 to 22).

Conventional farming practices were employed in the region, involving soil preparation (one plowing, two harrowings, and opening of planting furrows). The cassava cuttings material was planted with approximately 16 to 18 cm in length.

All field trials were conducted under rainfed conditions (without supplementary irrigation), following local crop management practices, in accordance with the recommendations of Souza et al. (27). Planting was carried out during the rainy season of the region (May to August) to ensure the minimum soil moisture required for germination and crop establishment.

Harvesting of the trials was done manually between 10 to 12 months after planting. The following root quality traits were evaluated: 1) dry matter content (DMC, %) in roots based on the gravimetric method: approximately 5 kg of roots were cleaned to remove excess soil, and their tips were trimmed. Then, the weight of roots in air and in water was measured, and DMC was calculated using the formula: $TMS = 158{,}3 \times \frac{Peso\ no\ ar}{Peso\ no\ ar - Peso\ na\ água} - 142$, as described by Kawano et al. (28); 2) starch content (SC, %) in roots: Starch from 500 g of roots from different plants in the plot was extracted as described in section 2.2.; cyanogenic compounds content (HCN): determined using the picrate method (29).

2.2 Starch extraction

The set of cassava samples analyzed consisted mainly of genotypes with significant variation in cyanogenic compound content (HCN), as well as in relation to dry matter content (DMC) and starch content. For this analysis, a panel containing 281 cassava clones, including improved and local (unimproved) cultivars (Table S1), was selected. Most of these samples originated from Brazil.

Starch extraction followed the procedure outlined by Vasconcelos et al. (30). Cassava roots were chosen, washed, and peeled to eliminate any external debris or impurities. Next, the root samples were sliced into smaller fragments to ease processing. These fragments were then blended at a ratio of 500 g of root to 500 ml of cold water (~5°C), with processing alternating between 1 minute of blending, 1 minute of rest, and an additional 1 minute and 30 seconds of blending. The resulting blend underwent filtration using voile fabric and a sieve with a mesh size of 220 micrometers. Subsequently, 500 ml of cold water was added to the filtered mass, which was blended again for approximately 1 minute. The filtered mass was rinsed with 3 liters of chilled water. The liquid component was left to settle in a cold chamber (around ±5 °C) for 12 hours to facilitate starch sedimentation, separating the liquid from the solid starch. After this period, the supernatant was discarded, and the starch was lightly washed with 20 ml of 95% alcohol. Following this,

the starch was dried in an oven at approximately ±40 °C for 3 days. Finally, the dried starch underwent a maceration process using a mortar and pestle until it reached a fine powder consistency. The starch powder was then stored in airtight containers, adequately shielded from moisture and potential sources of contamination, at approximately ±5 °C.

2.3 Evaluation of starch paste properties

The starch paste properties were assessed using a rapid viscosity analyzer (model RVA-4500, series 4; Newport Scientific, Warriewood, Australia), employing Standard 1 programming on Thermocline for Windows (TCW) software, version 7 (NewPort Scientific).

The analysis was conducted in duplicate using 3 g of starch sample (±14% moisture) in 25 g of distilled water. Correction of the samples and water weights to achieve 14% moisture content was performed using an infrared moisture analyzer (Gehaka IV2500). For this, the following formulas were applied: $m2 = \frac{(100-14)\times m1}{(100-u1)}$ and $w = 25,0 + (m1 - m2)$, where $m1$ is the mass of the sample in grams; $m2$ is the mass of the sample corrected to 14% basis in grams; $u1$ is the moisture content of the sample in %; and w is the corrected water weight in grams.

The starch sample was added to the container with water and homogenized with the RVA helix to prevent lump formation. The starch suspension underwent the following temperature/time program: heating to 50 °C for 1 minute; heating from 50 to 95 °C at a rate of 6 °C per minute; holding at 95 °C for 2.5 minutes; cooling from 95 °C to 50 °C at a rate of 6 °C per minute. Finally, the sample was held at 50 °C for 2 minutes. The suspension was subjected to 960 rpm for the first 10 seconds of the test and 160 rpm for the remainder of the time. The total analysis duration was 13 minutes per sample replicate. The evaluated parameters included: pasting temperature in °C (PaT); peak viscosity in cP (PeV); minimum viscosity at 95 °C in cP (MiV); viscosity breakdown in cP (BrD) which is obtained by the formula BrD = minimum viscosity – peak viscosity; final viscosity in cP (FiV); and setback or retrogradation tendency in cP (SeB), which is obtained by the formula SeB = final viscosity – minimum viscosity.

2.4 Evaluation of amylose content

To determine the amylose/amylopectin ratio, the iodine colorimetric method (31) was employed, which is based on the light transmission through the complex formed by the reaction between amylose and

iodine. Starch granules were dispersed with ethanol and gelatinized with 1 N sodium hydroxide. An aliquot was acidified, and after reaction with iodine, the blue-colored complex was quantified by spectrophotometry at 620 nm. A standard curve was constructed using diluted and serially diluted solutions of standard amylose (CAS: 9005-62-7, Sigma-Aldrich, St. Louis, USA) and amylopectin (waxy starch extracted from cassava root with 0% amylose). Results were expressed as a percentage according to the formula: Amylose (%) $= \frac{y \times 0.1}{m}$,, where y represents the percentage of amylose, 0.1 is the mass of the sample in grams that should be weighed to obtain the same result as the calibration curve, and m is the mass of the sample in grams.

2.5 Data analysis

The amylose content and starch paste properties were subjected to analysis of variance (ANOVA) using the following linear model: $Y_{ik} = \mu + g_i + \epsilon_{ik}$, where Y_{ik} represents the phenotypic observation of genotype i in repetition k; μ is the overall mean; g_i is the fixed effect of genotype, and ϵ_{ik} is the random residual effect of genotype i in repetition k. The data related to root quality were subjected to mixed model analysis for estimating the Best Linear Unbiased Estimators (BLUEs), using the following model: $Y_{ijk} = \mu + g_i + l_j + a_k + (g \times l)_{ij} + (g \times a)_{ik} + (g \times l \times a)_{ijk} + \epsilon_{ij}$, where Y_{ijk} is the phenotypic observation of genotype i within location j and year k; μ is the overall mean, l_j is the random effect of location; a_k is the random effect of year; g_i is the fixed effect of genotype, $(g \times l)$ is the random effect of genotype-location interaction, ($(g \times a)$ is the random effect of genotype-year interaction, $(g \times l \times a)$ is the random effect of genotype-location-year interaction, and ϵ_{ijk} is the residual random effect of genotype i within location j and year k.

The Pearson correlation between amylose content and starch paste properties was analyzed using the corrplot package (32). Additionally, Principal Component Discriminant Analysis (DAPC) was performed using the adegenet package (33). The number of clusters based on root quality attributes and agronomic aspects was determined using successive K-means clustering with an increasing number of groups (k ranging from 2 to 15) after transforming the dataset by Principal Component Analysis using the find.clusters function of the adegenet package (33). The Bayesian Information Criterion (BIC) was used to define the ideal number of clusters and the most suitable clustering solution with the lowest BIC. After determining the most suitable number of groups to represent germplasm diversity, the dendrogram was

constructed using the circlize package (34). Boxplots were obtained using the ggstatsplot package (35) to visualize the distribution and differences between clusters, compare different groups or variables, identify patterns, and provide a quick overview of descriptive statistics of the dataset of the 281 cassava genotypes under analysis. All packages were loaded and analyzed using R software, version 4.2.3 (36).

The selection index based on the sum of ranks (37) and predefined weights was used to select genotypes with a better balance of amylose content and root quality, according to the formula: $\text{SI} = (\text{DMC} \times 10) + (\text{Tamido} \times 10) + (\text{HCN} \times -30)$ which refers to the sum of the BLUEs of each characteristic multiplied by their respective economic weights. Initially, genotypes were pre-selected based on their amylose content values, i.e., low (<12%) and high content (>25%). Additionally, the selection differential was calculated based on the difference between the overall mean for each group and the mean of the selected genotypes.

3. Results

3.1 Variation of genetic parameters of root quality, amylose, and paste properties

The distribution of BLUEs for root quality traits by population (genetic material origin) is presented in Figure 1. The range of variation of BLUEs for dry matter content was quite wide (27.06 to 41.02%), with an average of 33.74% (improved genotypes) and 34.54% (germplasm). The same trend was observed for starch content with a variation of 14.61 to 25.67% (average of 20.49% in improved genotypes and 22.18% in germplasm). For HCN, the range of variation was also quite high, ranging from 1.77 to 7.81, with similar averages between the two populations (5.19 and 5.51). Only for HCN, there was a bimodal distribution of genotypes, especially those belonging to germplasm, and a frequency of genotypes with higher HCN content. For the other traits, there was a distribution close to normality (Figure 1).

Insert Figure 1

Similarly to the previous traits, there was a wide phenotypic variation for amylose content and starch paste properties (Figure 2). The variation in amylose content ranged from 0.05 to 33.23%, with an

average of 19.8% in improved genotypes and 22.1% in germplasm. Based on the genotypes evaluated in this study, no cassava genotypes with amylose content ranging between 5 and 10% were identified.

Insert Figure 2

All parameters associated with paste properties showed high phenotypic variation among the evaluated genotypes, such as peak or maximum viscosity (ranging from 2836.5 to 6676.5 cP with a mean of 4413 cP in improved genotypes and 4313 cP in germplasm), minimum viscosity (ranging from 506.0 to 2501.5 cP with a mean of 1702 cP in improved genotypes and 1496 cP in germplasm), viscosity breakdown (ranging from 1636.5 to 4552.0 cP with a mean of 2711 cP in improved genotypes and 2817 cP in germplasm), final viscosity (ranging from 251.0 to 3699.5 cP with a mean of 2517 cP in improved genotypes and 2275 cP in germplasm), retrogradation tendency (ranging from 38 to 1516 cP with a mean of 815 cP in improved genotypes and 779 cP in germplasm), time to peak viscosity (ranging from 2.97 to 4.53 min with a mean of 3.67 min in improved genotypes and 3.94 min in germplasm), and pasting temperature (ranging from 64.88 to 75.2 °C with a mean of 69.7 °C in improved genotypes and 71.1 °C in germplasm) (Figure 2). The improved population exhibited, on average, lower amylose values.

According to the analysis of variance, there was a significant effect ($p < 0.01$) for all traits related to amylose content and paste properties (Table 2). Since the residual effect and coefficient of variation estimates were very low, the heritability estimates were quite high for all these characteristics ($H^2 \geq 0.95$).

Table 2. Analysis of variance, phenotypic mean, and broad-sense heritability of amylose content and paste properties in cassava starch samples.

Source of variation	DF	Mean square							
		PeV	MiV	BrD	FiV	SeB	TPV	PaT	Aml
Genotype	280	826170.8*	257239.2*	440669.6*	764476.5*	177495.5*	0.1*	6.2*	81.3*
Rep	1	3872.0	107.3	2690.3	76.3	364.5	0.0	0.0	1.2
Residue	296	23928.0	3760.0	17425.0	9011.0	2975.0	0.0	0.2	1.8
H^2		0.97	0.99	0.96	0.99	0.98	0.95	0.96	0.98
Mean		4325.6	1525.0	2800.6	2310.9	785.8	3.9	71.0	21.9
CV%		0.0	0.0	0.0	0.0	0.1	0.0	0.0	0.1

DF: degree of fredoom; PeV: Peak viscosity (cP); MiV: Minimum viscosity (cP); BrD: Viscosity breakdown (cP); FiV: Final viscosity (cP); SeB: Retrogradation tendency (cP); TPV: Time to peak viscosity (min); PaT: pasting temperature (°C); Aml: Amylose content (%)

3.2 Phenotypic correlations

Positive phenotypic correlations between root quality traits, amylose content, and paste properties vary between 0.12 and 0.95, while negative correlations range from -0.14 to -0.41 (Figure 3). The highest positive correlations (>0.80) are observed between peak viscosity and viscosity breakdown, minimum viscosity and final viscosity, and final viscosity and retrogradation tendency. Moderate positive correlations (0.50 – 0.80) are evident for dry matter content versus starch content, peak viscosity versus minimum viscosity and final viscosity, and peak viscosity and minimum viscosity versus retrogradation tendency. Correlations between amylose content and paste properties are positive but of low magnitude (0.14 – 0.35), except for the correlation between amylose content and minimum viscosity, which is not statistically significant.

Insert Figure 3

Genotypes were categorized into two populations based on their status within the breeding program: those belonging to the germplasm bank (BAG) and those undergoing or having undergone improvement (improved population). Significant positive phenotypic correlations were observed within the germplasm for dry matter content and HCN, while a negative correlation was observed between starch content and amylose content (-0.18) (Figure 3). Correlations between these traits in improved genotypes did not reach significance, possibly due to the smaller sample size compared to the germplasm. In the improved population, correlations between amylose content and paste properties (PeV, MiV, BrD, FiV, SeB, and TPV) were stronger (0.60 – 0.86) compared to those observed in the germplasm.

Correlations between starch quality traits and amylose content were assessed considering genotypes' classification according to their HCN content (Figure 4). Genotypes classified as sweet cassava exhibited HCN content ranging from 2 to 4.5, while intermediate genotypes ranged between 4.6 and 6.0? and bitter genotypes ranged from 6.1 to 9. Moderate to high HCN content genotypes displayed significant positive correlations between dry matter content and starch content. However, in genotypes with low HCN

content (sweet), negative correlations were observed between dry matter content and amylose content (-0.23) and between starch content and amylose content (-0.27).

Insert Figure 4

To assess Pearson correlations among the evaluated traits, genotypes were categorized based on starch content (low < 19.60% and high > 19.60%) and dry matter content (low < 34.00% and high > 34.00%). In genotypes with low starch content, significant negative correlations were found between amylose content versus starch content (-0.55) and dry matter content (-0.39). Conversely, genotypes with high starch content exhibited significant but weak correlations (-0.17 to 0.24) with all paste property parameters (Figure 5). Despite variations in the starch content among the clones, the correlations between the parameters of paste properties remained consistent in both direction and magnitude, except for a few specific comparisons. For instance, correlations such as viscosity breakdown × HCN; time to peak viscosity × HCN, amylose content, minimum viscosity, final viscosity, and setback; paste temperature × peak viscosity, setback, and time to peak viscosity.

Insert Figure 5

Genotypes with higher dry matter content exhibited a significant correlation between dry matter content and HCN (0.22), although of low magnitude. However, in genotypes with lower dry matter content, significant correlations were identified between dry matter content × starch content (0.54), of moderate magnitude. In addition, genotypes with lower dry matter content were found to correlate with certain paste property traits, including peak viscosity (-0.26), minimum viscosity (-0.23), final viscosity (-0.23), setback (-0.21), and time to peak viscosity (-0.20), albeit with relatively low magnitudes.

Insert Figure 6

Figure 7 presents the network built based on the main significant correlations between paste properties, starch quality, and root quality. Overall, there was a clear separation between paste property and root quality traits. Additionally, strong relationships were identified between paste properties (peak

viscosity and viscosity breakdown, final viscosity and setback), as previously shown in the correlation analysis where the correlations between amylose content and paste properties were positive and of high magnitude in breeding populations (ranging from 0.60 to 0.86) compared to the correlations found in the germplasm. On the other hand, the paste temperature trait showed lower association with the other paste property attributes and root quality. Regarding starch quality, a positive association was found with paste properties, such as time to peak viscosity and viscosity breakdown.

Insert Figure 7

3.3 Discriminant analysis of principal components (DAPC)

DAPC was conducted to identify the genetic relationship among cassava genotypes based on phenotypic data of root quality, amylose content, and pasta properties (Figure 8). Three discriminant functions were retained, explaining 99% of the total genetic variation and allowing the grouping of genotypes into 6 clusters, consisting of 32, 52, 31, 17, 25, and 23 genotypes.

Insert Figure 8

Figure 9 depicts the distributions of phenotypic data concerning root quality for the six groups identified through DAPC analysis. In Group 1, the predominant attributes were median amylose content, low starch, and dry matter content. Group 2 exhibited median levels of amylose, dry matter, and starch content, alongside elevated levels of cyanogenic compounds. Group 3 displayed the widest data variation for both amylose content and cyanogenic compounds. While Group 4 featured median levels of amylose, starch, and dry matter content, it showed the lowest mean and variation in cyanogenic compound content. Group 5 was typified by low amylose content, yet high levels of starch and dry matter. Lastly, Group 6 encompassed genotypes with median amylose and dry matter content, but notably high levels of starch content.

Insert Figure 9

When analyzing the boxplots representing paste properties, a clear differentiation emerges: Genotypes within Group 1 are notably distinguished by their higher final viscosity, greater retrogradation tendency, and elevated pasting temperatures (Figure 10). In contrast, genotypes in Group 2 tend to exhibit lower average values for maximum, minimum, and final viscosity. Meanwhile, Group 3 stands out for its median viscosity values across maximum, minimum, and final measurements, accompanied by a lower viscosity breakdown and a notable retrogradation tendency, along with higher pasting temperatures.

Insert Figure 10

Genotypes within Group 4 exhibited notably higher values across various parameters (excluding the improved cultivars), including maximum viscosity, minimum viscosity, viscosity breakdown, final viscosity, and lower pasting temperature, compared to other groups. In contrast, genotypes in Group 5 demonstrated, on average, lower values of minimum viscosity, final viscosity, time to reach peak viscosity, and retrogradation tendency (averaging 733.37 cP). Conversely, Group 6 was distinguished by lower maximum viscosity, viscosity breakdown, and higher pasting temperature. The majority of genotypes from the breeding population were predominantly clustered within Groups 3 and 4, indicating that these genotypes were grouped due to genetic enhancements, resulting in higher average starch content (averaging 20.40%) and dry matter content (averaging 34.32%). The RVA profile of the six groups identified by DAPC is illustrated in Figure 11, providing insight into the average viscoamylogram of each group.

Insert Figure 11

3.4 Selection of genotypes based on amylose content

Extremes in amylose content are desirable due to their distinct industrial applications. Therefore, the selection of 15 genotypes with high and low amylose content for recombination in the cassava breeding program was performed. Most of the selected genotypes belong to the species germplasm, except for the clones BR-19F1wx-054-29 and BR-19F2wx-356-2 (Table 3). In the preliminary selection of genotypes with low amylose content (<12%), five genotypes with the highest index selection were ranked. The selection differential of the selected genotypes results in an increase in dry matter content (0.19%) and starch (2.06%), and a reduction in HCN by 0.26 compared to 1.53 in genotypes with higher amylose

content. On the other hand, for genotypes with the highest amylose content (>25%), 10 genotypes were ranked with an average of 27.18% amylose (range from 24.98% to 29.95%). The selection differential of genotypes with high amylose content allows for an increase in dry matter content (1.23%) and starch (1.54%), as well as a reduction in HCN (1.53) (Table 3).

Table 3. List of genotypes selected for recombination aiming to generate cassava progenies with low and high amylose content based on selection index, as well as the best linear unbiased estimator (BLUEs) and selection differential of the selected clones.

Genotype	Population	DMC	HCN	StC	Rank (Amylose < 12%)
BGM-0480	Germplasm	35.67	3.80	26.57	1
BGM-1042	Germplasm	34.26	3.66	25.28	2
BR-19F1wx-054-29	Improved	31.84	4.97	24.16	3
BGM-1834	Germplasm	30.81	4.52	23.36	4
BR-19F2wx-356-2	Improved	32.99	5.55	18.80	5
**Differential of selection**		0.19	-0.26	2.06	
**Genotype**	**Population**	**DMC**	**HCN**	**StC**	**Rank (Amylose > 25%)**
BGM-2358	Germplasm	34.04	2.42	25.24	1
Aipim-Guanambi	Germplasm	35.36	1.77	21.12	2
BGM-1313	Germplasm	35.57	3.16	22.53	3
BGM-1106	Germplasm	36.58	5.36	27.28	4
BGM-1148	Germplasm	36.84	4.83	24.80	5
BGM-1454	Germplasm	36.27	4.11	22.95	6
2019g-08-53	Germplasm	35.52	3.43	21.59	7
BGM-0998	Germplasm	35.99	4.18	22.90	8
BGM-0070	Germplasm	35.70	4.14	22.56	9
BGM-1044	Germplasm	34.77	3.31	20.71	10
**Differential of selection**		1.23	-1.53	1.54	

DMC: Dry matter content (%); HCN: Cyanogenic compound content; StC: Starch content (%).

4. Discussion

4.1 Genetic variability for root quality traits and amylose content in cassava

Estimating phenotypic variation in cassava germplasm is crucial for determining usable genetic variability and assessing potential selection gains. In this study, we observed a wide range of variation in root quality traits, amylose content, and paste properties, which offers opportunities for selecting genotypes suitable for commercial use or as parents in breeding programs.

Concerning root quality traits, the evaluated germplasm exhibited significant variability in dry matter content, ranging from 27.05% to 41.01%, with a mean of 34.75%. These findings align with those reported by Sánchez et al. (10) and Vasconcelos et al. (30), who found variations in dry matter content from 14.28% to 48.12% and from 28.01% to 39.70%, respectively, in germplasm accessions of *M. esculenta*. Therefore, while environmental factors strongly influence dry matter content (38), our results indicate a substantial genotypic contribution to this trait's variability.

Similarly, starch content in the roots exhibited considerable variation, ranging from 14.61% to 25.66%, with mean values of 22.18% and 20.49% in the germplasm and breeding population, respectively. These findings are consistent with those of Teixeira et al. (20), who observed starch content variations from 12.96% to 25.92% with a general mean of 19.47% across various cassava genotypes. The strong correlation between dry matter and starch content in cassava roots, as reported by Vasconcelos et al. (30) and Fukuda et al. (29), suggests similar distributions of these traits, despite the correlation not being perfect.

In the context of cyanogenic compounds, the germplasm analyzed encompassed genotypes categorized as sweet (HCN < 4.50), intermediate (4,5 > HCN < 6.00), and bitter (HCN > 6.00) according to Fukuda et al. (29). On average, the population exhibited intermediate levels of HCN, ranging from 5.19 to 5.51. Cyanogenic compound content holds particular importance in the development of sweet cassava varieties, predominantly consumed in cooked form, where roots should contain less than 100 mg kg^{-1} of HCN to ensure safety in consumption (39).

The variability in amylose content may influences the quality and texture of starch-based foods. Thus, understanding the correlation between paste properties and amylose content in cassava is pivotal for

guiding genetic improvement programs. Assessing diverse populations exhibiting wide-ranging amylose content is crucial for precisely delineating the relationship between paste properties and for developing more promising industrial starches (6). Amylose content displays notable variability among various cassava genotypes (8) and serves a critical role in evaluating the quality of products where starch is a primary component (8,40,41). In this study, amylose values ranged from 0.05 to 33.23%, a broader range than that reported by Teixeira et al. (20), whose variation spanned from 13.29% to 29.91%. Unlike our findings, where amylose content was higher among germplasm bank genotypes (22.1% versus 19.8% in the breeding population), Sánchez et al. (10) reported an average amylose content of 20.7%, with no significant discrepancy between local varieties and clones from the breeding program. In their study, amylose content varied from 15.02% to 26.5%. These differences in results may stem from various factors, including the varieties examined, growth conditions, and laboratory techniques employed.

4.2 Genetic variability for paste properties

The viscosity profile of starch is characterized by distinct stages, including paste formation, cooking, and retrogradation (15). Viscosity is pivotal in characterizing the rheological properties of various products, serving as a measure to evaluate a substance's ability to deform or resist deformation when subjected to tension (42). Starch paste viscosity is measured over time using RVA, providing insights into gelatinization capacity, paste stability, and other attributes relevant to starch processing (43).

Our study revealed significant phenotypic variation among genotypes in all assessed paste properties. This variability aligns with previous findings on cassava starch (10,14–19). Sánchez et al. (10) conducted an extensive investigation into starch paste characteristics, evaluating over 4,000 cassava genotypes from CIAT and identified considerable variability in starch paste properties, particularly maximum viscosity and viscosity breakdown.

In another study, Santos et al. (17) assessed 1031 cassava accessions for phenotypic variation in breeding, focusing on starch paste properties for food applications. They observed pasting temperature variation ranging from 64.1 °C to 74.1 °C. While Sánchez et al. (10) reported an average pasting temperature of 65.3 °C, our study found a slightly higher average (71.1 °C) for both the germplasm and breeding population (69.7 °C). This variance holds significance, offering potential benefits in the food industry by showcasing a broader range compared to conventional cultivars (63.4 °C to 66.9 °C) (17). The low pasting temperature indicate low resistance to swelling and easy formation of paste which makes it

more suitable for both food and non-food industrial processes because of a decrease in energy costs during production (19).

Starch retrogradation involves the rearrangement of gelatinized starch molecules, leading to the formation of organized crystalline structures. This process is highly relevant in starch processing, alongside its thermal properties, directly impacting the texture, stability, and other attributes of products incorporating starch as an ingredient (8). In this study, the average retrogradation tendency was measured at 785.8 cP, with a range spanning from 38 to 1516 cP. This value appears notably high when compared to data reported by Sánchez et al. (10) (ranging from -702 to 273 cP) and Onitilo et al. (19) (ranging from 19 to 80 cP). In contrast, Santos et al. (11) provided specific retrogradation tendency values for each group of evaluated cassava accessions. They examined 881 cassava genotypes from the Brazilian germplasm bank, along with their industrial potential using cassava and waxy maize clones. Notably, two genotypes exhibited a low retrogradation tendency, indicating a high capacity to maintain sensory quality and prolong the shelf life of refrigerated or frozen processed foods. Overall, starches with lower retrogradation tendency are preferred as they can uphold the texture and quality of foods for extended durations (44).

4.3 Correlations between root quality traits, amylose content, and paste properties

Investigating correlations between root quality traits, amylose content, and paste properties is important for guiding the selective breeding process of new cassava varieties. These correlations enable a deeper understanding of the relationship between root quality traits, especially those dependent on more labor-intensive laboratory analyses such as amylose content and paste properties (6).

In this study, the correlation between root quality traits in a breeding population and a set of germplasm was analyzed. Significant correlations were identified in two groups of clones, one from the breeding population and the other from the germplasm, including a positive relationship between starch content and dry matter (0.547 for the germplasm population and 0.591 for the breeding population) and a negative relationship between amylose content and starch content (-0.178 for the germplasm population and -0.222 for the breeding population). In the breeding population, amylose content showed significant correlations with paste properties, with medium magnitude for peak viscosity (0.610) and minimum viscosity (0.706), and high magnitude for other properties (>0.810), except for pasting temperature where

there was no significant correlation. There was a positive relationship between minimum viscosity and peak viscosity (0.129 in the germplasm population), but of low magnitude, while for retrogradation and final viscosity, the correlation of minimum viscosity showed high magnitude (0.795 and 0.957 respectively).

Genotypes with intermediate and high levels of HCN showed significant and medium-magnitude correlations between dry matter and starch content (0.798 and 0.687, respectively). Negative correlations of medium magnitude were observed between viscosity breakdown and pasting temperature, as well as viscosity breakdown and peak viscosity in genotypes with both high and low starch content. Additionally, there was a low-magnitude positive correlation for peak viscosity and minimum viscosity (0.244), final viscosity (0.286), and retrogradation (0.308).

Amylose content correlated with almost all parameters associated with paste properties, except pasting temperature in the breeding population, indicating a faster pasting process, reflecting in reduced cooking time for genotypes with higher amylose content (20). In contrast, the germplasm population showed correlations between amylose content only with peak viscosity.

High correlations were identified between maximum viscosity and viscosity breakdown (0.789 in the germplasm population and 0.986 in the breeding population), possibly due to starch structure and its behavior during processing or cooking, as amylose content directly affects gel formation and stability during cooking (43). Final viscosity and retrogradation tendency are related to changes in starch characteristics after cooking and cooling; that is, starches with higher amylose content tend to have higher final viscosity and greater retrogradation tendency after cooling, which is relevant for starch-containing food products as it affects texture and consistency after cooling and storage (7,8). Meanwhile, pasting temperature demonstrated a weaker association with other pasta property attributes and root quality traits.

4.4 Clustering of genotypes based on root quality traits, amylose content, and paste properties

The segregation of cassava genotypes into specific groups can be useful in plant breeding and allocating specific uses in the industry. Correlations between amylose content and paste properties are important as they directly affect food quality and texture (6). Genotypes in Group 5 have lower final viscosity compared to other groups, making them suitable for foods requiring stability during freezing. On the other hand, genotypes in Group 4 exhibit higher final viscosity, likely due to increased hydrogen

bonding interactions, and amylose molecules' higher tendency for reassociation, which also favors retrogradation (6).

The starch of genotypes in Group 1 shows a higher pasting temperature and thus can be used in applications requiring stability and resistance to high temperatures during processing or cooking (43). Conversely, Groups 1 and 6 show lower retrogradation tendency, making them more suitable for applications where minimizing or avoiding starch crystal formation after cooling is desired (8,16).

Most genotypes from the breeding population were predominantly grouped in Groups 3 and 4, comprising genotypes that underwent a breeding process resulting in higher starch and dry matter content (average of 20.40% and 34.32%, respectively). Additionally, Group 4 exhibited higher values for various parameters such as maximum viscosity, minimum viscosity, final viscosity, and lower pasting temperature compared to other groups (69.4°C). Furthermore, genotypes in Group 4, on average, showed higher viscosity parameters and lower pasting temperature compared to other groups. Therefore, it can be inferred that Group 4 presents characteristics related to paste properties, such as viscosity and pasting temperature, which may be relevant for the production of cassava-derived foods and products, making it an important group to advance the breeding program.

5. Conclusion

The phenotypic variability and correlations between amylose content and starch paste properties of different cassava genotypes were analyzed. The results demonstrated that improved genotypes showed more efficient and consistent outcomes regarding paste properties, providing promising potential for the production of cassava-derived foods and products with desired characteristics. This advancement in understanding the relationship between amylose content, starch content, dry matter, and paste properties can significantly contribute to the agricultural and agro-industrial sector, enabling more precise selection of cassava genotypes to meet the demands of the food and processing industry.

Conflicts of interest

The authors assert that they conducted the research without any financial or business relationships that may be perceived as a potential conflict of interest.

Funding

Natalia Rocha Ribeiro: CAPES (Coordenação de Aperfeiçoamento de Pessoal de Nível Superior). Grant number: 88887.641163/2021-00; Massaine Bandeira e Sousa: Empresa Brasileira de Pesquisa Agropecuária. Grant number: 20.18.01.012.00.00; Eder Jorge de Oliveira: CNPq (Conselho Nacional de Desenvolvimento Científico e Tecnológico). Grant number: 409229/2018-0, 442050/2019-4 and 303912/2018-9; Eder Jorge de Oliveira: FAPESB (Fundação de Amparo à Pesquisa do Estado da Bahia). Grant number: Pronem 15/2014. This work was partially funded by UK's Foreign, Commonwealth & Development Office (FCDO) and the Bill & Melinda Gates Foundation. Grant INV-007637. Under the grant conditions of the Foundation, a Creative Commons Attribution 4.0 Generic License has already been assigned to the Author Accepted Manuscript version that might arise from this submission. The funder provided support in the form of fellowship and funds for the research, but did not have any additional role in the study design, data collection and analysis, decision to publish, nor preparation of the manuscript.

Data Availability Statement

All datasets generated for this study can be found in the article and supplementary material

Author Contributions

Conceptualization L.A.O. and E.J.O.; methodology N.R.R. and L.A.O.; formal analysis N.R.R. and M.B.S.; investigation N.R.R.; resources E.J.O.; data curation N.R.R., M.B.S., and L.A.O.; writing—original draft preparation N.R.R.; writing—review and editing M.B.S., L.A.O., and E.J.O.; supervision M.B.S., L.A.O., and E.J.O.; project administration E.J.O.; funding acquisition E.J.O. All authors have read and agreed to the published version of the manuscript.

6. References

1. Ceballos H, Davrieux F, Talsma EF, Belalcazar J, Chavarriaga P, Andersson MS, et al. Carotenoids. In: Cvetkovic DJ, Nikolic GS, editors. IntechOpen; 2017, Available from: https://www.intechopen.com/chapters/55393

2. Parkes E, Aina O, Kingsley A, Iluebbey P, Bakare M, Agbona A, et al. Combining ability and genetic components of yield characteristics, dry matter content, and total carotenoids in provitamin A Cassava F_1 cross-progeny. Agronomy. 2020;10(12):1850. doi:10.3390/agronomy10121850
3. Agama-Acevedo E, Flores-Silva PC, Bello-Perez LA. Cereal starch production for food applications. In: Clerici MTPS, Schmiele. M, editors. Starches for Food Application. Elsevier; 2018. pp. 71–102. Available from: http://www.scopus.com/inward/record.url?scp=85081909672&partnerID=8YFLogxK
4. Zhang Y, Li G, Wu Y, Yang Z, Ouyang J. Influence of amylose on the pasting and gel texture properties of chestnut starch during thermal processing. Food Chem. 2019; 294: 378–83. https://doi.org/10.1016/j.foodchem.2019.05.070
5. Carmo, CD, Bandeira e Sousa, M, Pereira, JS, Ceballos, H, de Oliveira, EJ. Identification of waxy cassava genotypes using fourier□transform near□infrared spectroscopy. Crop Science, 2020;883–95. https://doi.org/10.1002/csc2.20102
6. Biduski B, Silva WMF da, Colussi R, Halal SL de ME, Lim LT, Dias ÁRG, et al. Starch hydrogels: The influence of the amylose content and gelatinization method. Int J Biol Macromol. 2018;113:443–9.
7. Palabiyik İ, Toker OS, Karaman S, Yildiz Ö. A modeling approach in the interpretation of starch pasting properties. J Cereal Sci. 2017;74:272–8. https://doi.org/10.1016/j.ijbiomac.2018.02.144
8. Tappiban P, Sraphet S, Srisawad N, Wu P, Han H, Smith DR, et al. Effects of cassava variety and growth location on starch fine structure and physicochemical properties. Food Hydrocoll. 2020;108:106074. https://doi.org/10.1016/j.foodhyd.2020.106074
9. Castanha N, Rojas ML, Augusto PED, Castanha N, Rojas ML, Augusto PED. An insight into the pasting properties and gel strength of starches from different sources: effect of starch concentration. Sci Agropecu. 2021;12(2):203–12. http://dx.doi.org/10.17268/sci.agropecu.2021.023
10. Sánchez T, Salcedo E, Ceballos H, Dufour D, Mafla G, Morante N, et al. Screening of starch quality traits in cassava (*Manihot esculenta* Crantz). Starch - Stärke. 2009;61(1):12–9. https://doi.org/10.1002/star.200800058
11. Santos TB dos, Carvalho CWP de, Oliveira LA de, Oliveira EJ de, Villas-Boas F, Franco CML, et al. Functionality of cassava genotypes for waxy starch. Pesqui Agropecuária Bras. 2021;56:e02414. https://doi.org/10.1590/S1678-3921.pab2021.v56.02414
12. Sakurai YCN, Rodrigues AM da C, Pires MB, Silva LHM da. Quality of pasta made of cassava, peach palm and golden linseed flours. Food Sci Technol. 2019;40:228–34.

13. Chisenga SM, Workneh TS, Bultosa G, Alimi BA. Progress in research and applications of cassava flour and starch: a review. J Food Sci Technol. 2019;56(6):2799–813. https://doi.org/10.1007/s13197-019-03814-6

14. Alamu EO, Maziya-Dixon B, Dixon AG. Evaluation of the pasting characteristics of cassava roots grown in different locations in Nigeria from the Genetic Gain Assessment trial. Front Sustain Food Syst 2022;6. Available from: https://www.frontiersin.org/articles/10.3389/fsufs.2022.1012410.

15. Awoyale W, Sanni LO, Shittu TA, Adegunwa MO. Effect of varieties on the functional and pasting properties of biofortified cassava root starches. J Food Meas Charact. 2015;9(2):225–32. doi:10.1007/s11694-015-9227-6

16. da Costa Nunes E, Uarrota VG, Moresco R, Maraschin M. Physico-chemical profiling of edible or sweet cassava (*Manihot esculenta Crantz*) starches from Brazilian germplasm. Food Biosci. 2021;43:101305. https://doi.org/10.1016/j.fbio.2021.101305

17. Santos CS dos, Sousa MB e, Brito AC, Oliveira LA de, Carvalho CWP, Oliveira EJ de. Phenotypic diversity of starch pasting properties in cassava for food industry. Acta Sci Agron. 2022;45:e56361. https://doi.org/10.4025/actasciagron.v45i1.56361

18. Seerangan K, Sheela MN, S.Ganeshan, Joel J. The Physicochemical, Biochemical and Pasting properties of forty one cassava (*Manihot esculenta* Crantz.) Landraces. Vegetos. 2013;27:76–85. doi 10.5958/j.2229-4473.27.1.013

19. Onitilo M, Sanni L, Daniel I, Maziya-Dixon B, Dixon A. Physicochemical and functional properties of native starches from cassava varieties in Southwest Nigeria. Journal of Food, Agriculture and Environment. 2007;5:108–14. ttps://biblio.iita.org/documents/onitilo-physicochemical-2007.pdf-8b7ccc1e51c1b3e99dbb895ab3fb07fd.pdf

20. Teixeira PRG, Viana AES, Cardoso AD, Moreira GLP, Matsumoto SN, Ramos PAS. Physical-chemical characteristics of sweet cassava varieties. Rev Bras Ciênc Agrár. 2017;12(2):158–65. doi:10.5039/agraria.v12i2a5433

21. Wang X, Reddy CK, Xu B. A systematic comparative study on morphological, crystallinity, pasting, thermal and functional characteristics of starches resources utilized in China. Food Chem. 2018;259:81–8. https://doi.org/10.1016/j.foodchem.2018.03.121

22. Ceballos H, Sánchez T, Morante N, Fregene M, Dufour D, Smith AM, et al. Discovery of an amylose-free starch mutant in cassava (Manihot esculenta Crantz). J Agric Food Chem. 2007;55(18):7469–76. doi 10.1021/jf070633y
23. Ascheri DPR, Boêno JA, Bassinello PZ, Ascheri JLR. Correlation between grain nutritional content and pasting properties of pre-gelatinized red rice flour. Rev Ceres. 2012;59:16–24. https://doi.org/10.1590/S0034-737X2012000100003
24. Karakelle B, Kian-Pour N, Toker OS, Palabiyik I. Effect of process conditions and amylose/amylopectin ratio on the pasting behavior of maize starch: A modeling approach. J Cereal Sci. 2020;94:102998. https://doi.org/10.1016/j.jcs.2020.102998
25. dos Santos TPR, Leonel M, Garcia ÉL, do Carmo EL, Franco CML. Crystallinity, thermal and pasting properties of starches from different potato cultivars grown in Brazil. Int J Biol Macromol. 2016;82:144–9. https://doi.org/10.1016/j.ijbiomac.2015.10.091
26. Yan S, Li Z, Wang B, Li T, Li Z, Zhang N, et al. Correlation analysis on physicochemical and structural properties of sorghum starch. Front Nutr, 2023;9. Available from: https://www.frontiersin.org/articles/10.3389/fnut.2022.1101868.
27. Souza L da S, Farias ARN, Mattos PLP de, Fukuda WMG. Livro Aspectos Socioeconômicos e Agronômicos da Mandioca. Cruz das Almas: Embrapa Mandioca e Fruticultura; 2006. 817 p. Available from: https://www.ciodaterra.com.br/aspectos-soc-e-agronomicos-da-mandioca
28. Kawano K, Fukuda W, Cenpukdee U. Genetic and environmental effects on dry matter content of cassava root. Crop Sci. 1987;27:69–74. https://doi.org/10.2135/cropsci1987.0011183X002700010018x
29. Fukuda CMG, Guevara Gómez CL, Kawuki RS, Ferguson ME. Selected morphological and agronomic descriptors for the characterization of cassava. International Institute of Tropical Agriculture; 2010. Available from: https://cgspace.cgiar.org/handle/10568/88053.
30. Vasconcelos LM, Brito AC, Carmo CD, Oliveira PHGA, Oliveira EJ. Phenotypic diversity of starch granules in cassava germplasm. Genet Mol Res GMR. 2017;16(2). doi: 10.4238/gmr16029276
31. International Organization for Standardization. ISO 6647-1: Rice — Determination of amylose content — Part 1: Spectrophotometric method with a defatting procedure by methanol and with calibration solutions of potato amylose and waxy rice amylopectin. 1987. Available from: https://global.ihs.com/doc_detail.cfm?document_name=ISO%206647%2D1&item_s_key=00467585

32. Wei T, Simko V. R package "corrplot": Visualization of a Correlation Matrix. (Version 0.92) Available from: https://rdrr.io/cran/corrplot/

33. Jombart T, Devillard S, Balloux F. Discriminant analysis of principal components: a new method for the analysis of genetically structured populations. BMC Genet. 2010;11:94. http://www.biomedcentral.com/1471-2156/11/94

34. Guo Y, Li Y, Hong H, Qiu LJ. Establishment of the integrated applied core collection and its comparison with mini core collection in soybean (*Glycine max*). Crop J. 2014;2(1):38–45. https://doi.org/10.1016/j.cj.2013.11.001

35. Patil I. Visualizations with statistical details: The "ggstatsplot" approach. 20 de fevereiro de 2024 [cited 20 february 2024]; Available from: https://osf.io/p7mku

36. R Core Team. R: The R Project for Statistical Computing [Internet]. 2023 [cited 20 february 2024]. Available from: https://www.r-project.org/

37. Mulamba NN, Mock J. Improvement of yield potential of the ETO blanco maize (*Zea mays* L.) population by breeding for plant traits. Egypt J Genet Cytol. 1978;1:40-51. Available from: https://www.semanticscholar.org/paper/Improvement-of-yield-potential-of-the-ETO-blanco-by-Mulamba-Mock/8be6eba42c0e7d62b1a19535be64255aeb337b35

38. Oliveira EJ, Oliveira Filho OS, Santos VS. Classification of cassava genotypes based on qualitative and quantitative data. Genet Mol Res GMR. 2015;14(1):906–24. http://dx.doi.org/10.4238/2015.February.2.14

39. Fuhrmann E, Vieira EA, Fialho J de F, Faleiro FG, Malaquias JV, Carvalho LJCB. Agronomic and biochemical evaluation of cassava clones with roots that have pink pulp. Ciênc Rural. 2020;50:e20180910. https://doi.org/10.1590/0103-8478cr20180910

40. Ahmed S, Zhou X, Pang Y, Xu Y, Tong C, Bao J. Genetic diversity of potato genotypes estimated by starch physicochemical properties and microsatellite markers. Food Chem. 2018;257:368–75. https://doi.org/10.1016/j.foodchem.2018.03.029

41. Lemos PVF, Barbosa LS, Ramos IG, Coelho RE, Druzian JI. Characterization of amylose and amylopectin fractions separated from potato, banana, corn, and cassava starches. Int J Biol Macromol. 2019;132:32–42. https://doi.org/10.1016/j.ijbiomac.2019.03.086

42. Zortéa MEB, Demiate IM, Praxedes MA, Wosiacki G. Avaliação da viscosidade aparente de pastas de amidos nos viscosímetros Brookfield RVDV-II+ Pro e Rápido Visco-Analisador RVA-4. Rev Bras Tecnol Agroindustrial, 2011;5(1). Available from: doi: 10.3895/S1981-36862011000100008
43. Williams G, Ansah KO, Agbenorhevi JK, Oduro IN, Bobobee E. Characterization of starch from new cassava accessions at different maturity. J Food Secur. 2019;7(5):170–4. doi:10.12691/jfs-7-5-3
44. Diamantino VR, Costa MS, Taboga SR, Vilamaior PSL, Franco CML, Penna ALB. Starch as a potential fat replacer for application in cheese: Behaviour of different starches in casein/starch mixtures and in the casein matrix. Int Dairy J. 2019;89:129–38. https://doi.org/10.1016/j.idairyj.2018.08.015

Captions

Figure 1. Distribution of best linear unbiased estimator (BLUEs) of dry matter content (DMC, %), starch (StC, %), and cyanogenic compounds (HCN) in cassava genotypes. Red color: genotypes belonging to the improved population; Green color: genotypes belonging to the germplasm bank.

Figure 2. Frequency distribution of amylose content and various paste properties among cassava genotypes. Genotypes from the improved population are represented in red, while those from the germplasm bank are in green.

Figure 3. Pearson correlation by population (Pop1: germplasm; Pop2: improved population) among root quality traits, amylose content and paste properties. DMC: dry matter content (%); HCN: cyanogenic compounds content; StC: starch content (%); Aml: amylose content (%); PeV: peak viscosity (cP); MiV: minimum viscosity; BrD: viscosity breakdown (cP); FiV: final viscosity (cP); SeB: setback (cP); TPV: time to peak viscosity (min); PaT: paste temperature (°C). *, **, and *** indicate significant correlations ($p < 0.05$, $p < 0.01$, and $p < 0.001$, respectively).

Figure 4. Pearson correlation of root quality traits, amylose, dry matter content, and starch content in cassava genotypes based on classification of HCN content, that is bitter (Bit), sweet (Swe) and intermediate (Int). DMC: dry matter content (%); HCN: cyanogenic compounds content; StC: starch content (%); Aml: amylose content (%); PeV: peak viscosity (cP); MiV: minimum viscosity (cP); BrD: viscosity breakdown (cP); FiV: final viscosity (cP); SeB: setback (cP); TPV: time to peak viscosity (min); PaT: paste temperature (°C). *, **, and *** indicate significant correlations ($p < 0.05$, $p < 0.01$, and $p < 0.001$, respectively).

Figure 5. Pearson phenotypic correlation in cassava genotypes with high (Hig) and low (Low) starch content. DMC: dry matter content; HCN: cyanogenic compounds content; StC: starch content (%); Aml: amylose content (%); PeV: peak viscosity (cP); MiV: minimum viscosity (cP); BrD: viscosity breakdown (cP); FiV: final viscosity (cP); SeB: setback (cP); TPV: time to peak viscosity (min); PaT: paste temperature (°C). *, **, and *** indicate significant correlations ($p < 0.05$, $p < 0.01$, and $p < 0.001$, respectively).

Figure 6. Pearson correlation in cassava genotypes with high and low dry matter content. DMC: dry matter content; HCN: cyanogenic compounds content; StC: starch content; Aml: amylose content (%); PeV: peak viscosity (cP); MiV: minimum viscosity (cP); BrD: viscosity breakdown (cP); FiV: final viscosity (cP); SeB: setback (cP); TPV: time to peak viscosity (min); PaT: paste temperature (°C). *, **, and *** indicate significant correlations ($p < 0.05$, $p < 0.01$, and $p < 0.001$, respectively).

Figure 7. Regularized network of partial correlations among root quality traits, amylose content, and starch properties evaluated in cassava genotypes. The thickness of the lines represents the intensity of the correlations, while green and red colors represent positive and negative correlations, respectively. DMC: dry matter content (%); HCN: cyanogenic compounds content; StC: starch content (%); Aml: amylose content (%); PeV: peak viscosity (cP); MiV: minimum viscosity (cP); BrD: viscosity breakdown (cP); FiV: final viscosity (cP); SeB: setback (cP); TPV: time to peak viscosity (min); PaT: paste temperature (°C).

Figure 8. Discriminant analysis of principal components (DAPC) showing the genetic relationship of cassava genotypes considering phenotypic data on root quality traits, amylose content, and paste properties. Three components explain 99% of the total genetic variation. Groups determined by the *find.clusters* function.

Figure 9. Boxplot of different groupings of cassava genotypes for root quality-related characteristics. The bars represent comparisons of significant means ($p<0.05$).

Figure 10. Boxplot of the diverse groupings of cassava genotypes based on paste properties. The bars denote comparisons of statistically significant means ($p<0.05$).

Figure 11. Rapid visco analyser profile of different clusters obtained by discriminant analysis of principal components analysis of cassava genotypes based on paste properties.

Figure1

Click here to access/download;Figure;Figure1.tif

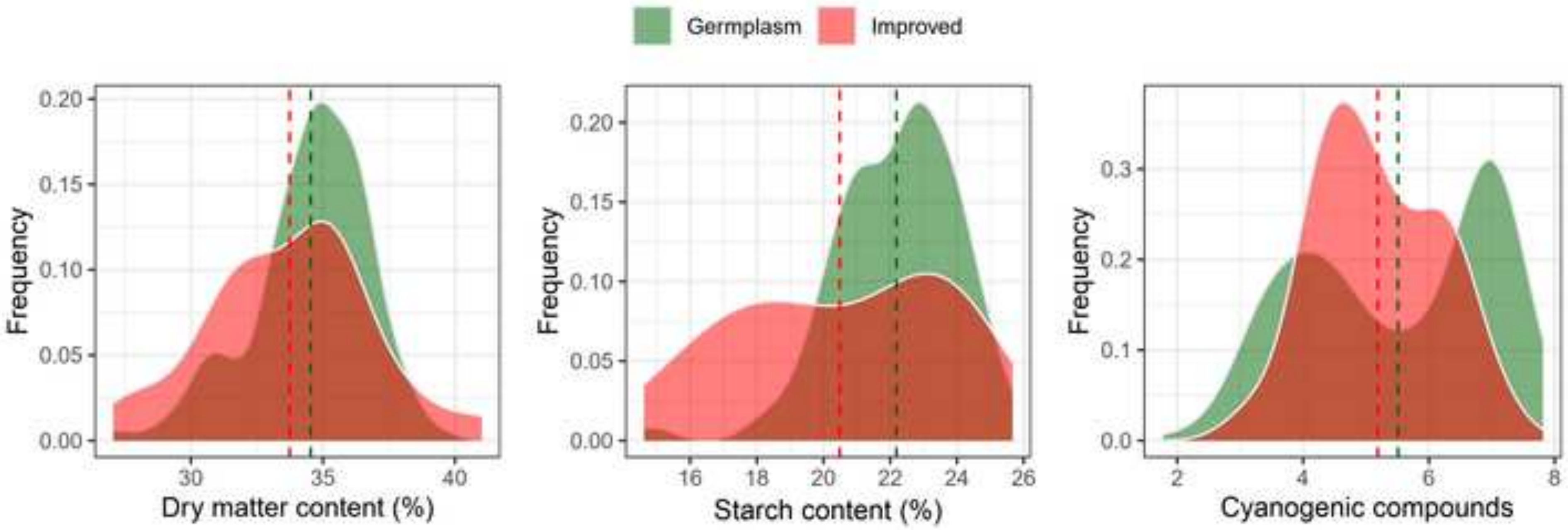

Figure2

Click here to access/download;Figure;Figure2.tif

Figure2.tif

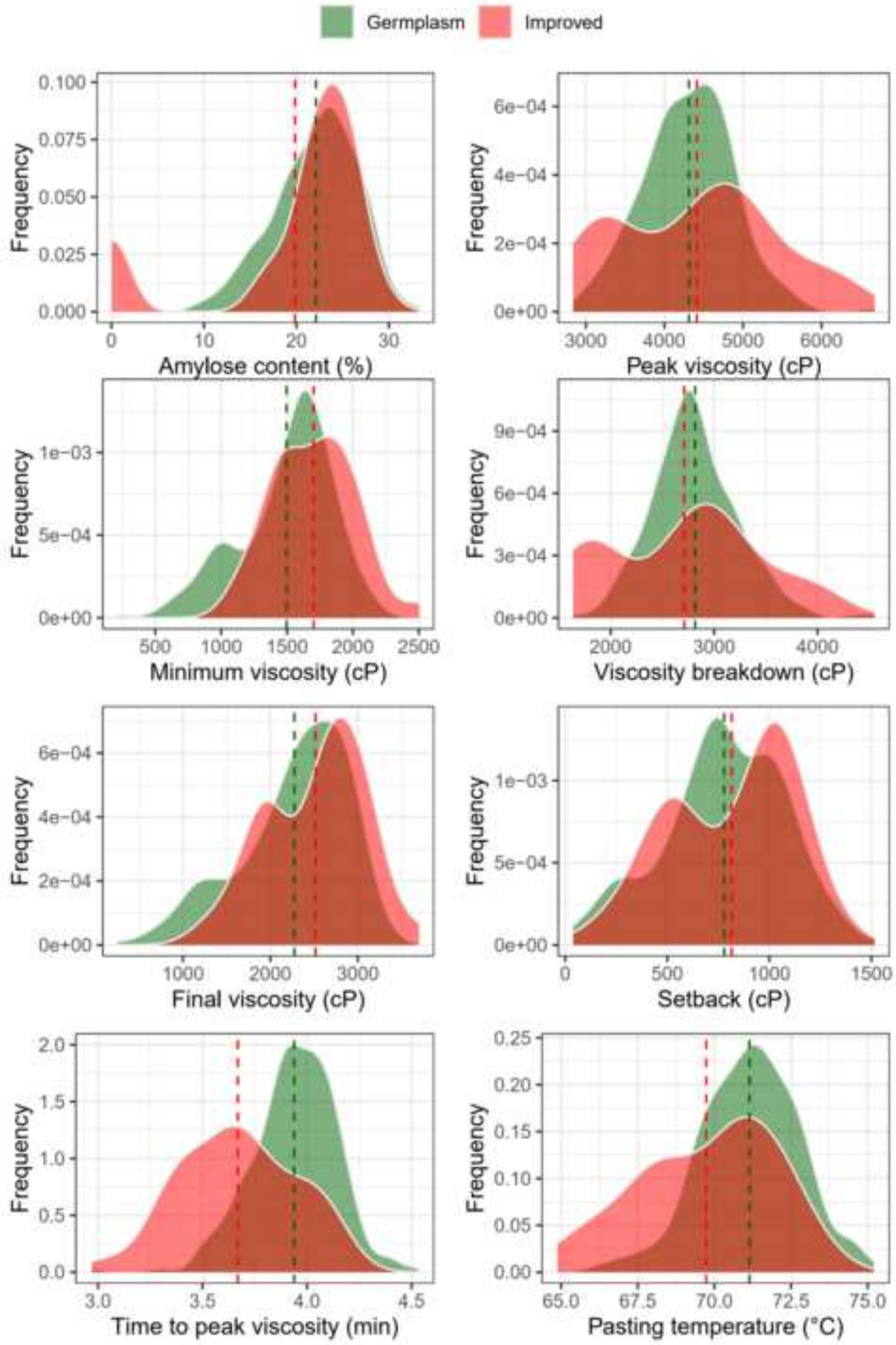

Figure3

Click here to access/download;Figure;Figure3.tif

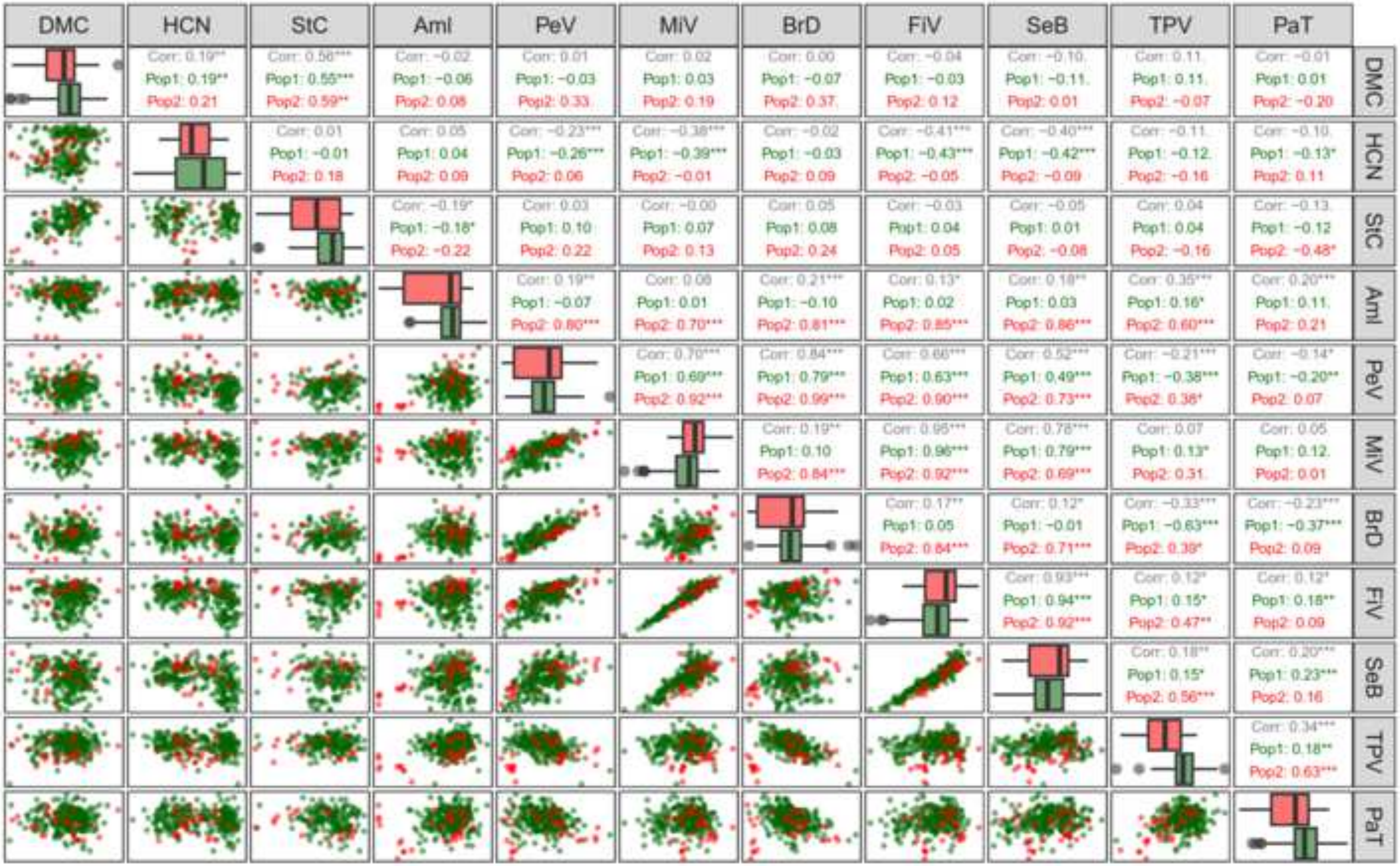

Figure3.tif

Figure4

Click here to access/download;Figure;Figure4.tif

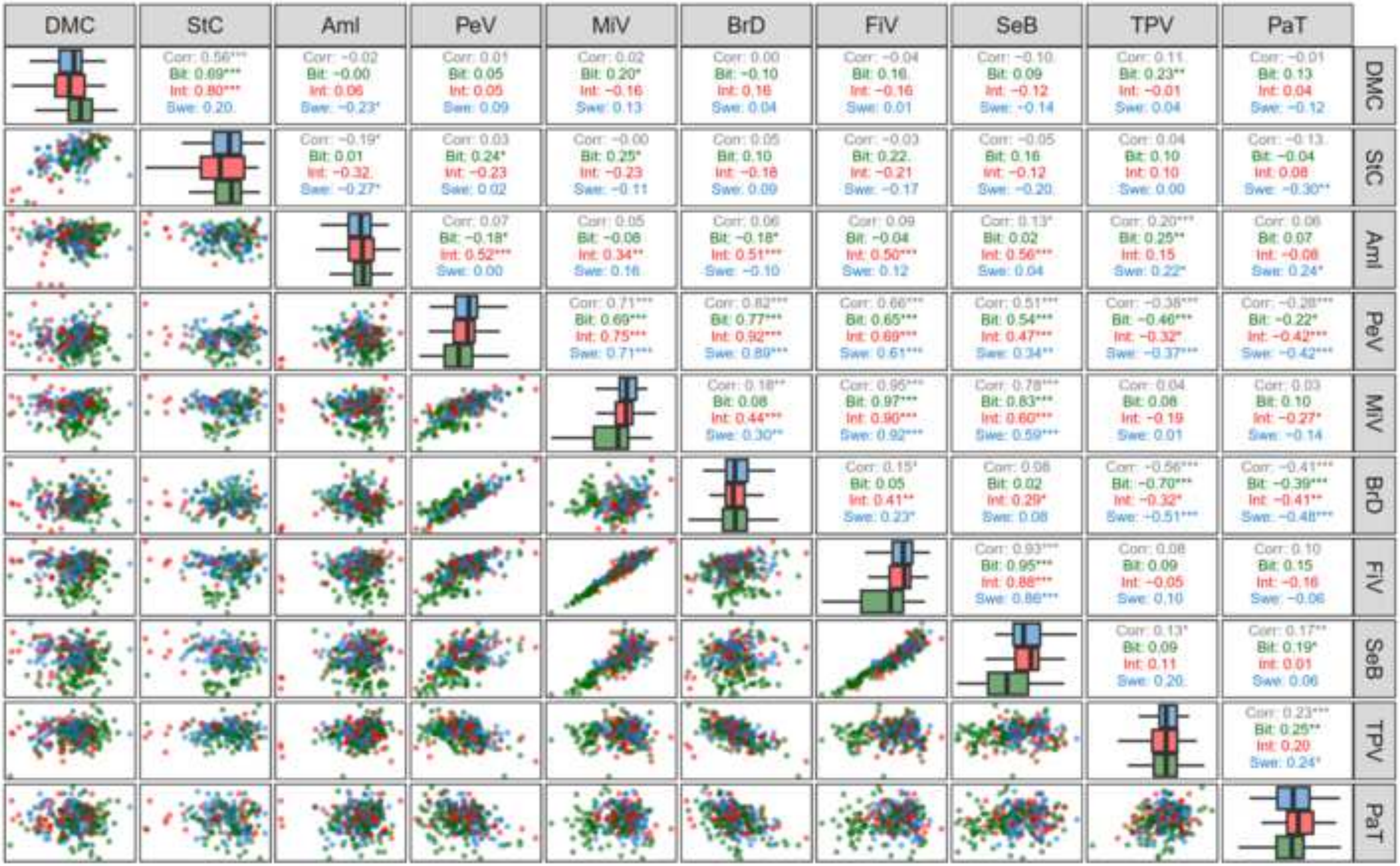

Click here to access/download;Figure;Figure5.tif

Figure5.tif

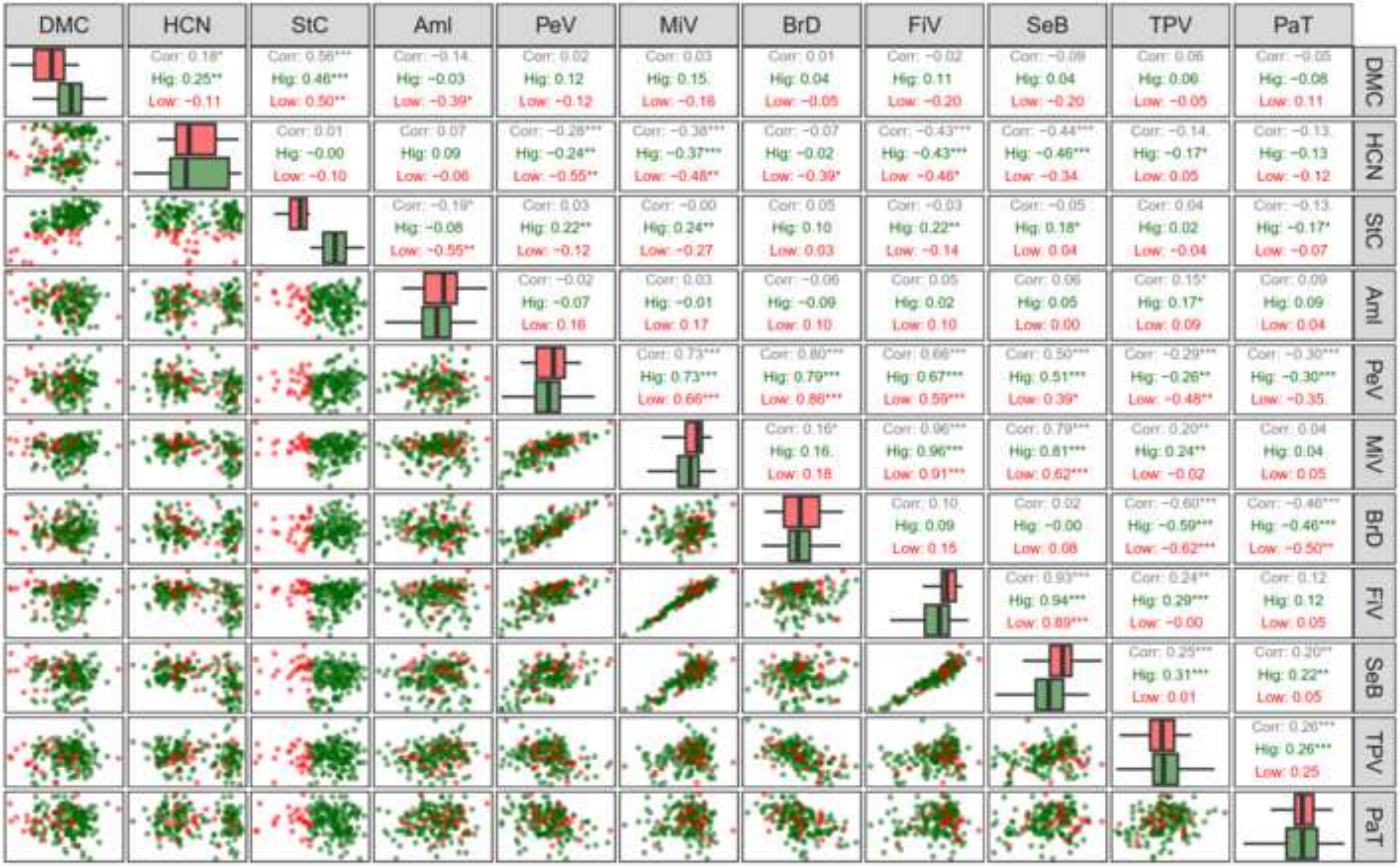

Figure6

Click here to access/download;Figure;Figure6.tif

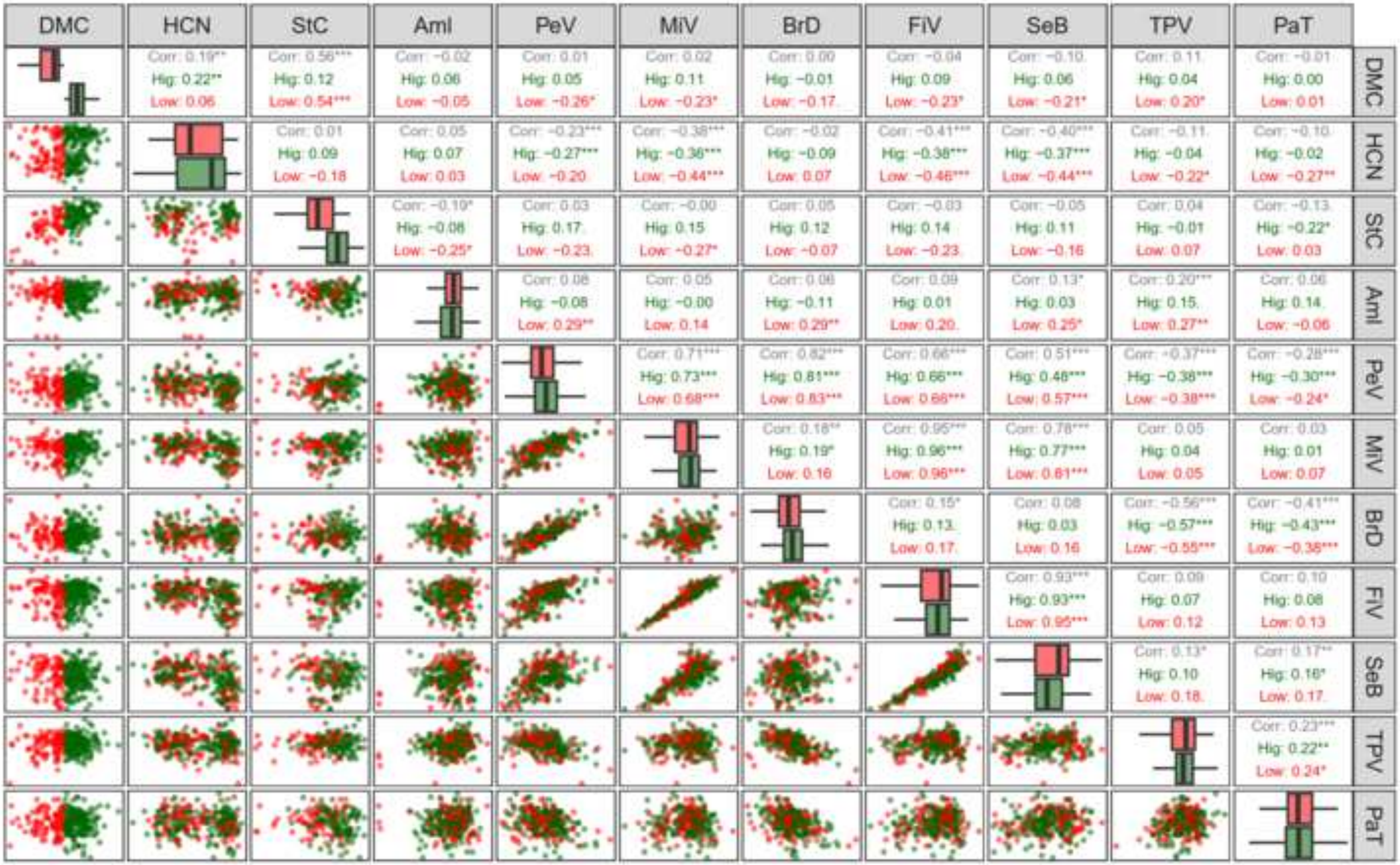

Figure6.tif

Click here to access/download;Figure;Figure7.tif

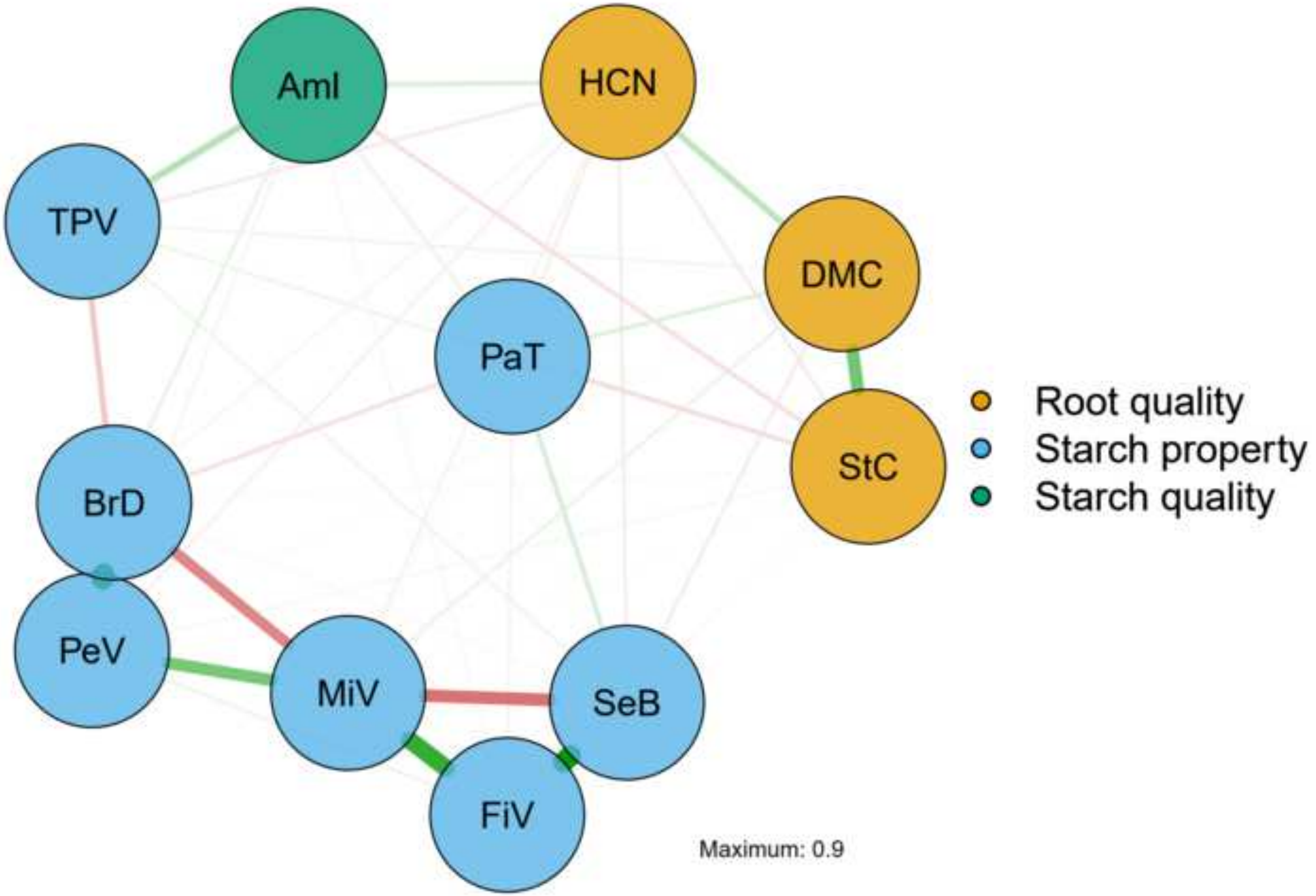

Click here to access/download;Figure;Figure8.tif

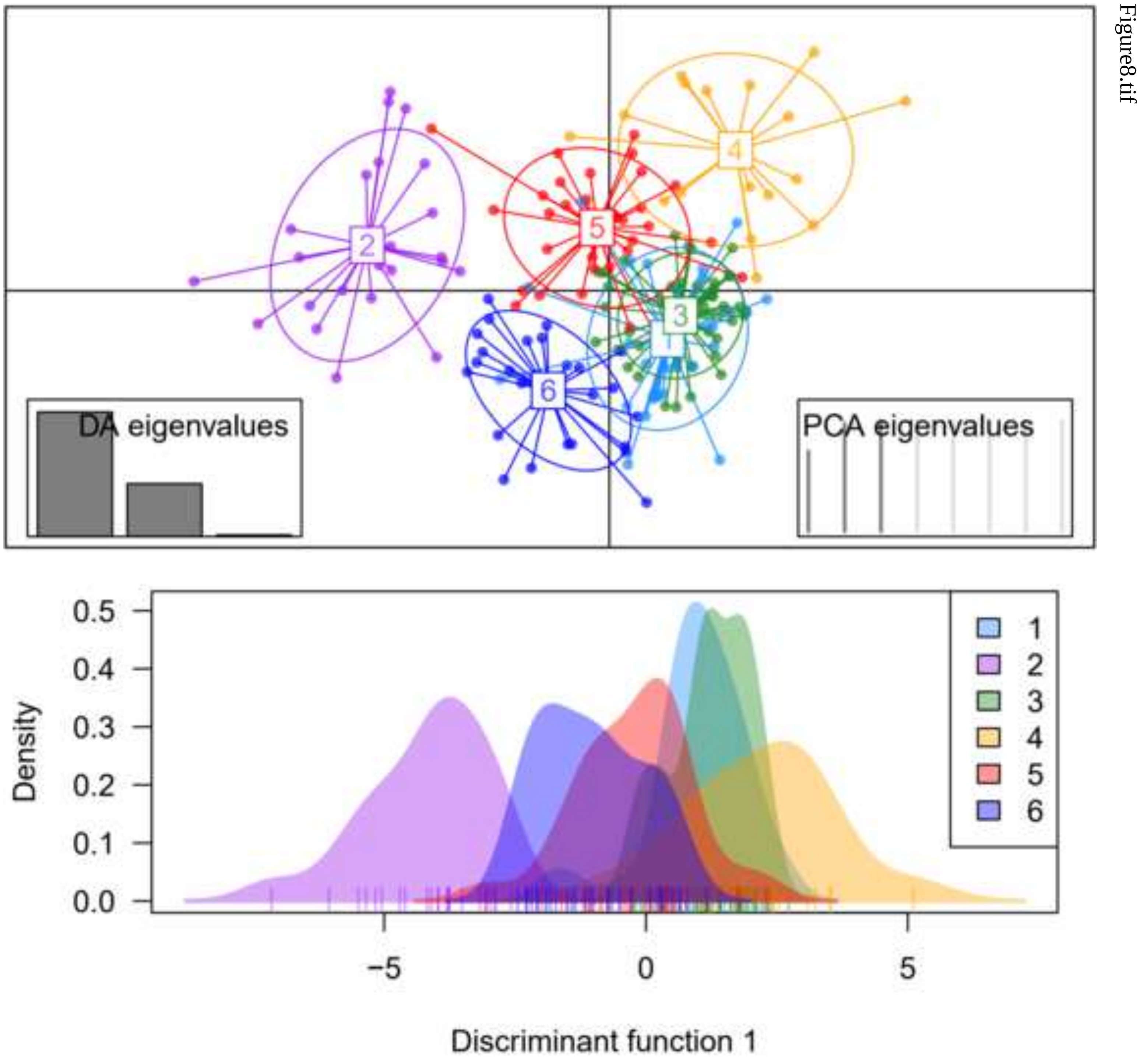

Figure9

Click here to access/download;Figure;Figure9.tiff

Figura9.tiff

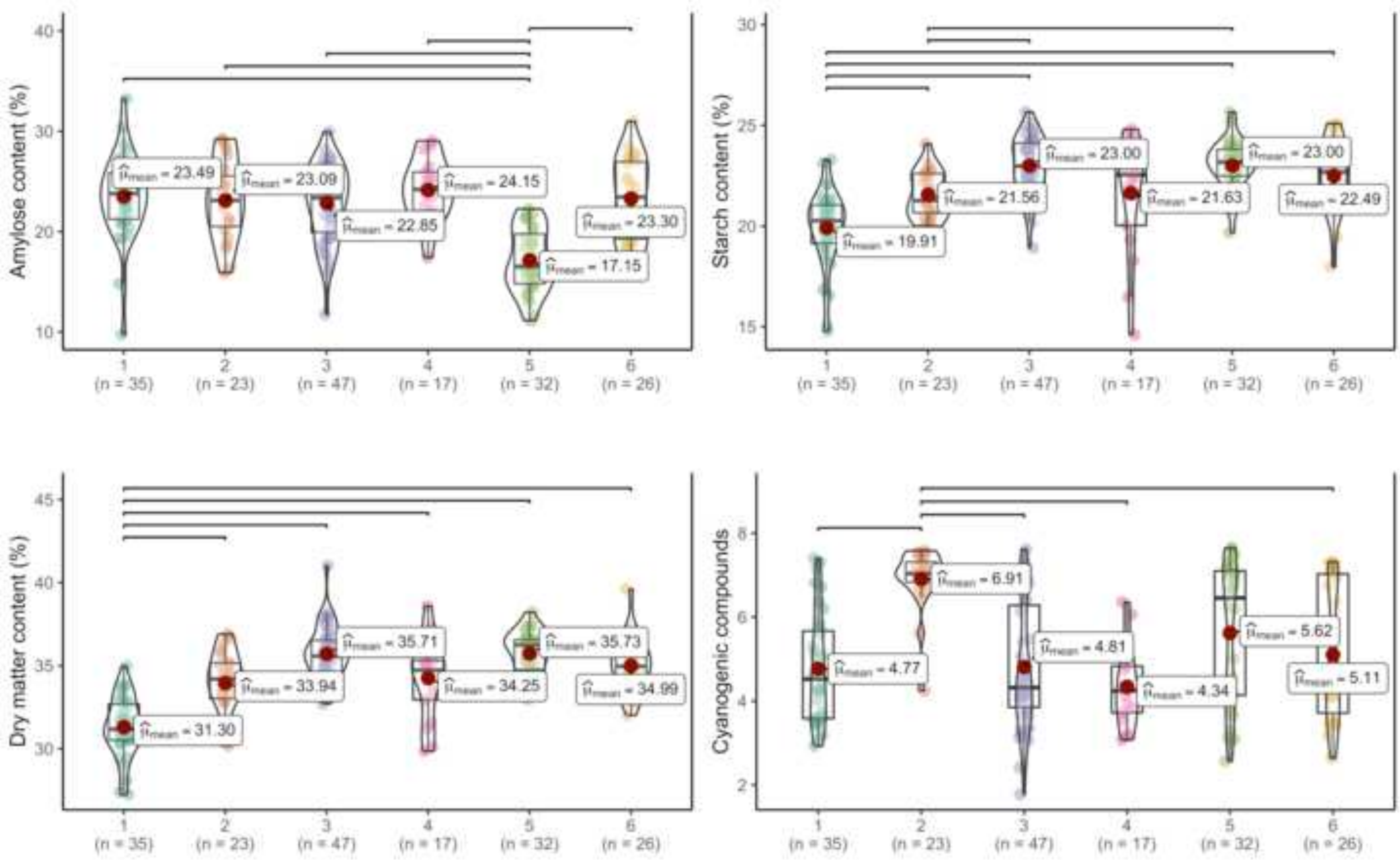

Figure10 Click here to access/download;Figure;Figure10.tiff

Figura10.tiff

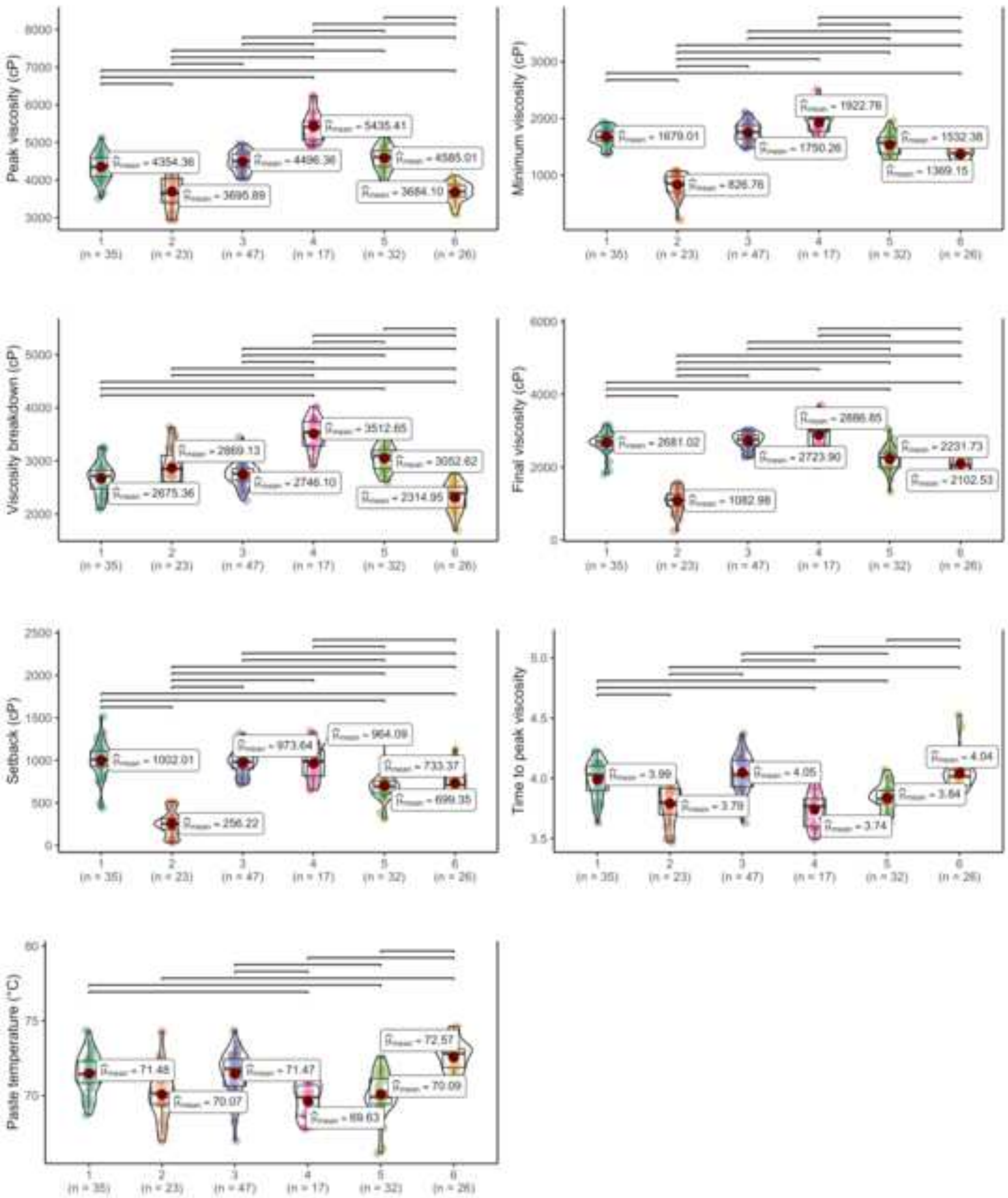

Figure11

Click here to access/download;Figure;Figure11.tif

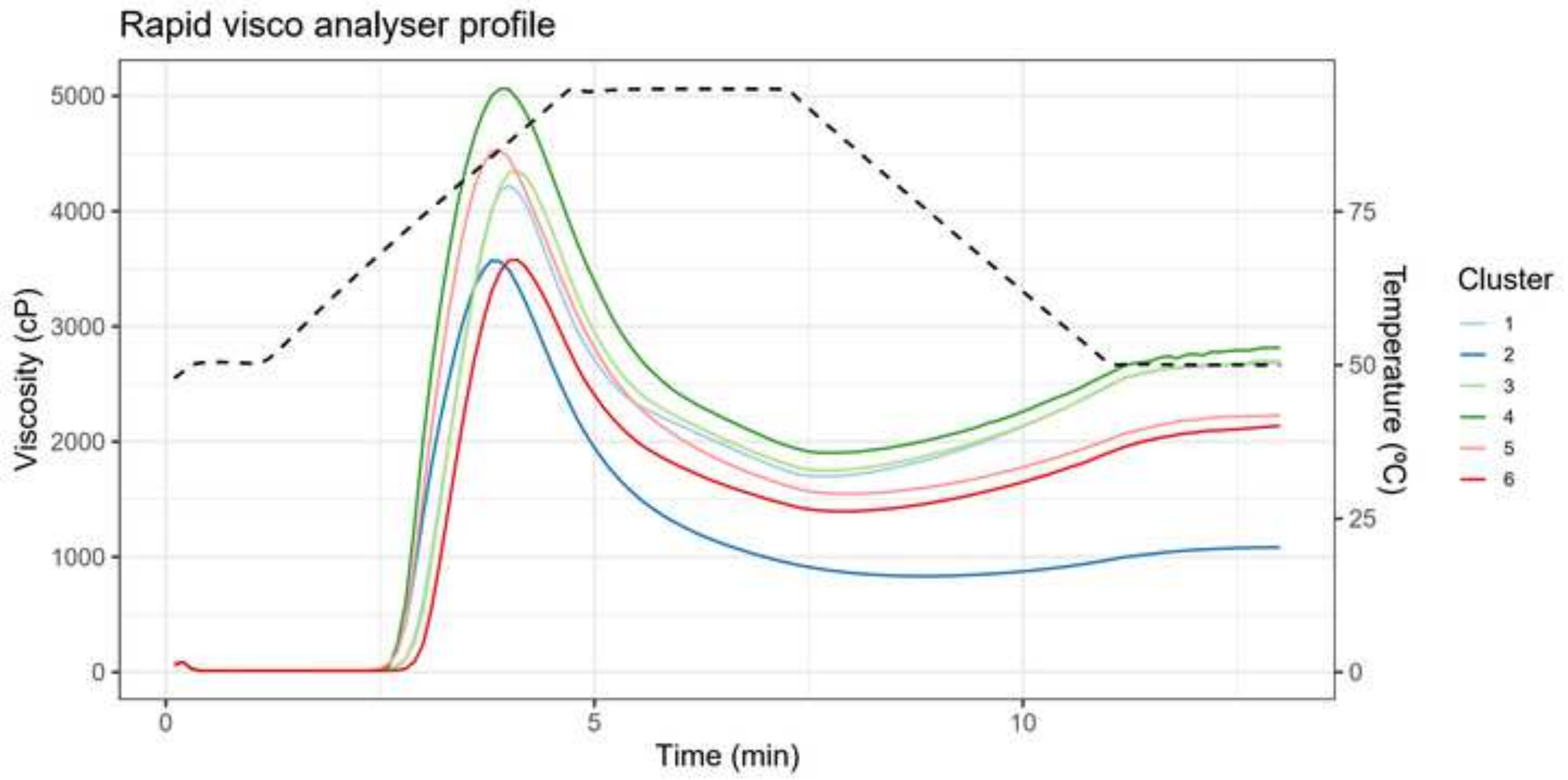

Figure11a.tif

Click here to access/download
Supporting Information
Ribeiro et al_PlosOne_2024 Table S1.docx
