# Supplementary material for: Variability of amylose content and its correlation with the paste properties of cassava starch
Source: PLoS One. 2024 Oct 23;19(10):e0309619. doi: 10.1371/journal.pone.0309619 (PMC11498679; doi:10.1371/journal.pone.0309619)
Supplement: Attachment — Submitted filename: Comments PLOS One VN Cassava.docx [file pone.0309619.s002.docx]

**Review Comments**

The objective of the article *Variability of amylose content and its correlation with the paste properties of cassava starch* was to examine the genetic diversity related to amylose content in the cassava germplasm bank and explore its correlation with the features of starch paste in a population that exhibits significant differences in root yield and characteristics.

To precisely define the relationship between amylose content and paste characteristics and to create more promising industrial starch types, extensive investigations involving varied populations exhibiting notable differences in amylose content are necessary. Therefore, the findings of this study can be helpful for further research, which makes the results significant for the scientific peers.

In the Introduction part of the manuscript, the subject was well addressed and the citation of the literature is adequate and up to date.

The Material and Methods are elaborately presented and the methods used are appropriate for this kind of research. A large number of trials, cassava genotypes, and analyses were performed which makes this study significant.

The Results are very elaborate, and the Discussion part of the article tackles the research subject appropriately, and discusses the findings in detail, by making appropriate conclusions and references to other previously conducted research. In the conclusion, the results are summarized in an adequate manner. The References are up to date and refer to the subject correctly.

I find the article suitable for publishing after minor revision.

These are my comments:

**Page 4, line 89**: Correlations between pasting properties and amylose content were found on wheat, as well (see <https://doi.org/10.1111/jfpp.15805>).

**Page 6, line 132**: Please translate the words in the formula into English.
