# Supplementary material for: Variability of amylose content and its correlation with the paste properties of cassava starch
Source: PLoS One. 2024 Oct 23;19(10):e0309619. doi: 10.1371/journal.pone.0309619 (PMC11498679; doi:10.1371/journal.pone.0309619)
Supplement: Attachment — Submitted filename: Response to reviewers.docx [file pone.0309619.s004.docx]

**PONE-D-24-09179**

**Variability of amylose content and its correlation with the paste properties of cassava starch**

**Reviewer 1:**

I find the article suitable for publishing after minor revision.

These are my comments:

**Page 4, line 89**: Correlations between pasting properties and amylose content were found on wheat, as well (see <https://doi.org/10.1111/jfpp.15805>).

**Response:** We have added the requested citation

**Page 6, line 132**: Please translate the words in the formula into English.

**Response:** Ok, thank you for letting us know about the need for the translation.

**Reviewer 2:**

All the minor changes requested in the PDF file have been made in the new document (in red).
